# Mechanism of Antioxidant Activity of Betanin, Betanidin and Respective C15-Epimers via Shape Theory, Molecular Dynamics, Density Functional Theory and Infrared Spectroscopy

**DOI:** 10.3390/molecules27062003

**Published:** 2022-03-21

**Authors:** Iliana María Ramirez-Velasquez, Ederley Velez, Alvaro Bedoya-Calle, Francisco Jose Caro-Lopera

**Affiliations:** 1 Faculty of Exact and Applied Sciences, Instituto Tecnológico Metropolitano ITM, Cll. 73 # 76A-354, Medellín 050034, Colombia; 2 Faculty of Basic Sciences, University of Medellin, Cra. 87 # 30-65, Medellín 050026, Colombia; evelez@udemedellin.edu.co (E.V.); ahbedoya@udemedellin.edu.co (A.B.-C.); fjcaro@udemedellin.edu.co (F.J.C.-L.)

**Keywords:** antioxidant mechanisms, DFT calculations, shape theory, molecular docking

## Abstract

Betanin and betanidin are compounds with extensive interest; they are effectively free radical scavengers. The present work aims to elucidate the differences between the mechanism of the antioxidant activity of betanin, betanidin, and their respective C15-epimers. Shape Theory establishes comparisons between the molecules’ geometries and determines parallelisms with the descriptors BDE, PA, ETE IP, PDE, and infrared spectra (IR) obtained from the molecule simulations. Furthermore, the molecules were optimized using the B3LYP/6-31+G(d,p) protocol. Finally, the molecular docking technique analyzes the antioxidant activity of the compounds in the complex with the therapeutic target xanthine oxidase (XO), based on a new proposal for the geometrical arrangement of the ligand atoms in the framework of Shape Theory. The results obtained indicate that the SPLET mechanism is the most favorable in all the molecules studied and that the first group that loses the hydrogen atom in the four molecules is the C17COOH, presenting less PA the isobetanidin. Furthermore, regarding the molecular docking, the interactions of these compounds with the target were favorable, standing out to a greater extent the interactions of isobetanidin with XO, which were analyzed after applying molecular dynamics.

## 1. Introduction

*Antioxidants* are chemicals that inhibit or delay unwanted oxidation reactions. They offer their electrons to free radicals and thus prevent cell damage. About the substrate subjected to oxidation, the antioxidant protects it from irreversible danger, preventing its oxidative conversion through reactive species. Thus, when antioxidant substances are present in food, they delay, control, or inhibit oxidation and deterioration of food quality [1,2,3]. Researches in recent years have focused on the search for species with high free radical-scavenging activity. In this line, there is evidence in the literature that some natural colorants, such as betalains, are compounds that act by inhibiting the action of free radicals that contribute to the appearance of several degenerative diseases [4]. As for pharmaceutical, cosmetic, and food applications, betalains are stable in a pH range of 3.5 to 7. This characteristic gives them the importance of being used in low acid and neutral products [5,6].

Betalains are derived from betalamic acid and are soluble in water. They consist of two groups of structures: betaxanthins, with yellow-orange coloration, and betacyanins, responsible for the red-purple color. Betaxanthins are immonium conjugate of betalamic acid with amino acids or amines, whereas betacyanins are immonium conjugate of betalamic acid with cyclo-DOPA, traditionally glycosylated. Although tyrosine is the precursor of this last element, in higher plants, the precursor of betalamic acid is 4,5-seco-DOPA, which constitutes the basic structure of the betalain skeleton [7]. Figure S1 of the Supplementary Material shows the essential structure representation of betacyanins.

The substitution via glycosylation or acylation of one or both hydroxyl groups located at positions 5 and 6 of this compound results in different betacyanin derivatives. Predominantly 5-O-glycosides and glycosylation of 5-O-glycoside and esterification with hydroxycinnamic acids are also common [7]. Thus, Betanin (betanidin-5-O-β-glucoside) is the most widespread betacyanin among plants; for example, red beet contains a concentration of betanin in the range of 300–600 mg/kg, and presents concentrations, in smaller amounts, of isobetanin (See Figure S2 in Supplemetary Material), betanidin, and betaxanthins [8,9].

Hydrolysis of Betacyanin produces Betanidin, and the epimer at C-15 produces Isobetanin or a mixture of the two aglycones. Moreover, their conjugated double bond is associated with their color, where the maximum light absorption at 540 nm is for the red Betacyanins [10].

When acting in biological systems, antioxidants produce changes in cellular elements’ chemical composition or structure. For instance, oxidation of xanthine takes place at the molybdopterin (Mo-pt) center, and the electrons thus introduced are rapidly distributed to the other centers by intramolecular electron transfer. The crystal structure of salicylate-XO was reported and provided for structure-based docking studies [11,12,13,14,15].

Several studies were developed and demonstrated the efficacy of betalains as free radical scavengers. For instance, it was demonstrated that the antioxidant capacity of betanin is pH-dependent; these results were analyzed from the calculation of phenolic OH homolytic bond dissociation energy (BDE) and ionization potential (IP). These parameters were obtained from DFT B3LYP/6-311+G** or B3LYP/6-31G** quantum-mechanical calculations level of theory [16].

The present work aims to extend the research in this line by approaching the modeling of betanin (Bn), isobetanin (IsoBn), betanidin (Bd), and isobetanidin (IsoBd) to point out how marked are the differences between the mechanism of the antioxidant activity of the molecules in question. The main reaction mechanisms HAT (hydrogen atom transfer), SPLET (sequential proton-loss electron transfer), and SET-PT (sequential electron-proton transfer) are analyzed in an aqueous medium to demonstrate which of them, from the thermodynamic point of view, is the most favorable mechanism for each molecule and to establish differences between the compounds studied concerning these mechanisms. For the achievement of this purpose, the named Shape Theory have used to establish similarities and dissimilarities between the molecules geometry and based on that to determine correspondences with the descriptors: BDE (bond dissociation enthalpy), related to the HAT mechanism, PA (proton affinity) and ETE (electron transfer enthalpy) associated with the SPLET mechanism, IP (ionization potential) and PDE (proton dissociation enthalpy) related to the SEP-PT mechanism. The theoretical study (DFT) achieves from the parent molecules’ structural geometries and their respective radical forms. Frontier orbitals (HOMO/LUMO) were also studied. Antioxidant activity of the betalains via implementing a formalism based on the distances between the molecules in the named Shape Theory, a subject included in mathematics and statistics, was analyzed.

Shape theory is a new area for comparing and estimating the average shape and variability between objects summarized by a finite number of reference points; a large list of applications can be found in various sciences [17]. The Gaussian statistics was used extensively [17,18]; now, robust models for shape theory are available for several transformations (affine, polar, SVD, QR) and for real normalized division algebras (real, complex, quaternion, and octonion) [19,20,21,22,23,24,25]. A crucial concept for our purposes, includes the Riemannian distance (RD) and was applied recently in the following fields: spectroscopic analysis [26] and rocking curves [27]; stochastic search of molecules [28]; and remote sensing of burning satellite products [29].

In the context of chemistry and the present paper, [28] sets the shape theory as a robust discrimination method for molecules, since it uses geometric invariances based on rigid Euclidean motions and measures the distances of molecules in equivalence class spaces. Instead of using the redundant methods based on random search and nongeometrical meaning changes of molecules for optimization, [28] proved the theoretical and computational efficiency of shape theory for finding non repeated equilibrium structures of nanoclusters, using their Riemannian geometry properties and considering the multiple local minima of the potential energy surface.

In the present work, we implement the shape theory in two new ways: firstly, as a discriminant method of optimized molecules and their antioxidant capacity, and secondly, as an alternative method for molecular docking based on the Riemannian geometry.

Another aspect considered is the set of IR spectra obtained from the simulations of the molecules under study, which allowed understanding the vibrational mechanisms of the molecules associated with their respective frequency and their correlation with the molecular descriptors associated with the antioxidant capacity.

IR spectra are a way to assess compounds’ chemical stability and physical capacity [30], with rapid quantification allowing the collection of specific molecular fingerprints from samples [31]. These capabilities and the shape theory allow a complete characterization of the compounds [26].

## 2. Methods

### 2.1. Dft Studies

The capacity to scavenge free radicals (R${}^{•}$) through a hydrogen atom transfer (H${}^{•}$) from phenolic hydroxyl groups to the free radical, either in one step or in two steps, was mainly approached through three mechanisms (i) HAT, a direct Hydrogen Atom Transfer between the antioxidant and the active radical (Equation (1)), (ii) SPLET (Sequential Proton Loss Electron Transfer): deprotonation of the antioxidant which resulting anion, followed by an electron transfer to the active radical, the final step is protonation of the anion of the active radical (Equation (2)) and (iii) SET-PT (Single Electron Transfer Proton Transfer): an electron is transferred from the antioxidant to the active radical, yielding a cation-radical and an anion, followed by proton transfer from the cation-radical to the anion (Equation (3)). These mechanisms may occur parallel but with different rates [11,29,32,33,34,35].

(1) $$\mathrm{ArOH}+R^{•}\to {\mathrm{ArO}}^{•}+\mathrm{RH}$$

(2) $$\mathrm{ArOH}+R^{•}\to {\mathrm{ArO}}^{-}+H^{+}+R^{•}\to {\mathrm{ArO}}^{-}+R^{•}+H^{+}\to {\mathrm{ArO}}^{•}+\mathrm{RH}$$

(3) $$\mathrm{ArOH}\to {\mathrm{ArOH}}^{•+}+R^{-}\to {\mathrm{ArO}}^{•}+\mathrm{RH}$$

Descriptions that characterize each one of the mechanisms are: the bond dissociation enthalpy (BDE) characterizes the HAT mechanism, the SLEPT mechanism via the proton affinity (PA), and the electron transfer enthalpy (ETE) and SET-PT mechanism via the ionization potential (IP), and the proton dissociation enthalpy (PDE).

The following equations calculate the descriptors:

(4) $$\mathrm{BDE}=H\left({\mathrm{ArO}}^{•}\right)+H\left(H^{•}\right)-H\left(\mathrm{ArOH}\right)$$

(5) $$\mathrm{PA}=H\left({\mathrm{ArO}}^{-}\right)+H\left(H^{+}\right)-H\left(\mathrm{ArOH}\right)$$

(6) $$\mathrm{ETE}=H\left({\mathrm{ArO}}^{•}\right)+H\left(e^{-}\right)-H\left({\mathrm{ArO}}^{-}\right)$$

(7) $$\mathrm{IP}=H\left({\mathrm{ArOH}}^{•+}\right)+H\left(e^{-}\right)-H\left(\mathrm{ArOH}\right)$$

(8) $$\mathrm{PDE}=H\left({\mathrm{ArOH}}^{•}\right)+H\left(H^{+}\right)-H\left({\mathrm{ArOH}}^{•+}\right)$$

where Hx is the enthalpy of the system at 298.15 K and a pressure of 1 atm.

The package Gaussian 09 conducted the calculations [36]. In addition, PubChem’s repository facilitates the download files of the structures of Betanin and Betanidin used for the DFT studies, and from them, the stereoisomers were obtained [37]. The free radical-scavenging activity can be determined via studies with different levels of theory and have reported that the computational level DFT B3LYP/631+G(d,p) has allowed analyzing the system quite well at a low computational cost [11,16,32,38].

The parent molecules and their respective radicals, radical cations, and anions were optimized at the B3LYP level of theory and with the 6 − 31 + G(d,p) basis set without any geometry constraints [39]. The radical or ionic structures were optimization starting from the parent molecules’ optimized geometries, applying the available method (UB3LYP(6 − 31 + G(d,p). The systems involving open-shell species are sensitive to spin contamination. Spin contaminations of radicals showed a value between 0.76–0.78. After annihilating the first spin contaminant, they dropped to the correct value of 0.75. Therefore, spin contamination should not bias found reaction enthalpies.

The solvation via an implicit method with Integral-Equation-Formalism Polarizable Continuum Model (IEFPCM) as the model [40,41]. At the calculated vibrational frequencies, no imaginary frequency modes were obtained, confirming the stability of the optimized structures.

The numerical values-calculated to determine the most favorable antioxidant mechanism from the Equations (4)–(8) of the theoretical descriptors: BDE, PA, ETE, IP, PDE, and the energy values corresponding to the frontier molecular orbitals HOMO/LUMO. All enthalpies-calculated at 298 K and 1.0 atm. Previous reports provide the enthalpies in the water of the hydrogen atom, proton, and electron: the enthalpy of the proton H(H+) is −259.00 kcal/mol, the enthalpy of the electron H(e−) is −55.61 kcal/mol, and the enthalpy of the hydrogen atom H(H−) is −314.65 kcal/mol [32,42]. The IR-simulated spectra via the Gaussian 09 package for the parent molecules Bn, Bd, IsoBn, and IsoBd. The energetic study of the loses the hydrogen atom mechanisms was conducted based on IR spectra and comparing the group frequencies and the fingerprint frequencies [43].

### 2.2. Shape Spaces

In the context of chemistry, [28] define the shape of a molecule as all geometric information that remains after eliminating rotation, translation, scaling, or reflection of the cluster. For a mathematical treatment, molecules of k atoms in three dimensions are writing as kx3 matrices. After removing translation, rotation, scaling, or reflection, the shape of the molecule is just a point in a matrix space. The so-called Riemannian distance (RD) is one of the most important descriptors in shape theory. [28] and the present work uses the RD to find the similarity or dissimilarity of two molecules from a given optimization process. In that sense both molecules can be seen as two points in a hypersphere, understood as the space of equivalence classes of invariant matrices under translation and scaling [17,18]. Thus, the RD is an angle in hypersphere, ranged from 0 (equal molecules) to π/2 (extremely different molecule), and it serves as an excellent nonredundant descriptor (from the Riemannian geometrical meaning) that classifies molecules. The RD is also sensitive to small changes of the atom positions, which is important for measuring subtle discrepancies in the chemical processes of antioxidant activity.

The first application of shape theory in the present work involves the characterization of the Riemannian distances between deprotonated molecules and the respective parent molecules.This discrimination is studied from two perspectives: (1) we compare the complete deprotonated molecules to observe the global distance due to the effect of antioxidant activity; (2) we study the discrepancy in the subspaces defined by each chemical element, as an unusual geometric property implicit in the optimization process. Both geometric measures, global and partial, are related to other purely chemical descriptors.

We make the calculations with own algorithms implemented in R, a free software environment for statistical computing and graphics [44]; the routines collect the Shape Theory concepts provided by [17], and the chemical applications inspired by [28].

### 2.3. A Shape Theory Docking Method and Molecular Dynamics

The second method derived from shape theory, involves a new docking approach. It considers the intrinsic and the invariant Riemannian geometrical information of the ligands. The molecular docking analysis pursues a model for the protein-ligand complex, in order to elucidate the interactions with the amino acid residues in the active site that allows the inhibition of the XO enzyme. Then, the supporting description via RD can be used for as a suitable explanation of the antioxidant capacity of these compounds.

The new molecular docking method was also analyzed by proposing a preferential system for the ligand coordinates, which is invariant under similarity transformations (rotations, scaling and translations). It considers the maximal spatial variability of the ligand. Although, the spectral decomposition theorem can provide one preferential system based on variability, the strict order of the atoms can be perturbed by reflections involved in the rotation matrices given by the principal components method. This method can produce molecules without a chemistry framework. Our well-behaved invariant method generalizes Bookstein’s coordinates of shape theory [17]. In our case, the method proposes the two most distant ligand atoms as the baseline. Then, the new transform is rotated in such way that maximal variability lies of the *x*-axis. A second rotation is performed to the second perpendicular maximal variability is on the *y*-axis. In a similar manner a third rotation places the new ligand in such way that the third orthogonal variability remain in the *z*-axis. This invariant ligand is called the template. We complete the method by proposing nine ligands which are rotations (90, 180, and 270 degrees) of the template, originally placed in the *xy*, *xz*, and *yz* planes. Finally, we perform the molecular docking of the original template a the nine new ligands inside the given protein.

The new method avoids the usual random search which does not distinguish equivalent classes of ligands. The usual methods are redundant allowing repetitions with no meaningful new geometrical and invariant information.

We explore the probable interactions of the union of the compounds with XO via molecular docking with the AutoDock Vina program [45,46]. For docking studies, the X-ray crystal structure of bovine XO in complex with salicylate (PDB ID code 1FIQ, Protein Data Bank: http://www.rcsb.org/pdb accessed on 16 December 2021) was used [47] in 21 June 2021. For the development of this study, we selected the enzyme xanthine oxidase XO, which is present in some mammalian tissues, in various species, from bacteria to humans. Its importance lies in the fact that it produces reactive oxygen species either alone or in combination with other enzymes and compounds. XO belongs to the group of oxidoreductases that catalyzes the oxidation of hypoxanthine to xanthine and xanthine to uric acid in the last stage of the metabolic degradation of purines, with the production of reactive oxygen species, as mentioned. This phenomenon is related to various pathological states such as chronic heart failure, cardiomyopathy in diabetes, and gout [12,13,33].

Ligand preparation and receptor one include the remotion of the A- and B-chains of the protein and all small molecules. First, we added the polar hydrogen atoms to the macromolecule file, and then a grid box was defined to enclose the active site with dimensions of 20 Å *×* 20 Å *×* 20 Å, and a center set at the point *x* = 26, *y* = 10, *z* = 118. Finally, the final docked structure for each compound was analyzed using the VMD visualization program [48]. It compares the results with those obtained from docking of the Febuxostat inhibitor with the XO active site [15,33].

We carried out the molecular dynamics (MD) study to simulate the local conformational changes in the complex resulting from the union between the highest-ranked compound in molecular docking and the XO enzyme. The procedure allows both the ligand and all the protein residues to reach a steady-state conformation.

The NAMD (Nanoscale Molecular Dynamics) [49] and CHARMM-GUI (Chemistry at Harvard Macromolecular Mechanics- GUI) [50] were used regarding computer tools. The CharmM36 force field was used, integrated into the graphical user interface for CHARMM, which can calculate parameters of bound or unbound molecules. On the other hand, the TIP3P water model was chosen. Then an environment was created to put the molecule together with the water. For this, the protein was placed in an orthorhombic system with the number of water molecules needed to solvate the box. The simulation was carried out in 10 ns. For the molecular dynamics, we were using the best conformation obtained from the molecular docking stage. After doing the docking, we can start with the Dynamics Molecular. On the other hand, to better analyze the complexes formed in the computational study, the VMD graphic viewer was used to obtain the last trajectory (frame) corresponding to 10 ns and the most energetically stable of the molecular dynamics carried out. Analyzed the trajectories obtained, and the interactions with the enzyme were obtained.

Another novelty of the new shape theory method proposed here, connects the Riemannian distance with results of docking summarized by the protonic affinity. This important fact provides an inusited relation with the geometry properties emerging from the Riemannian invariance of the protein-ligand complex.

## 3. Results and Discussion

### 3.1. DFT Studies

**Figure 1:**
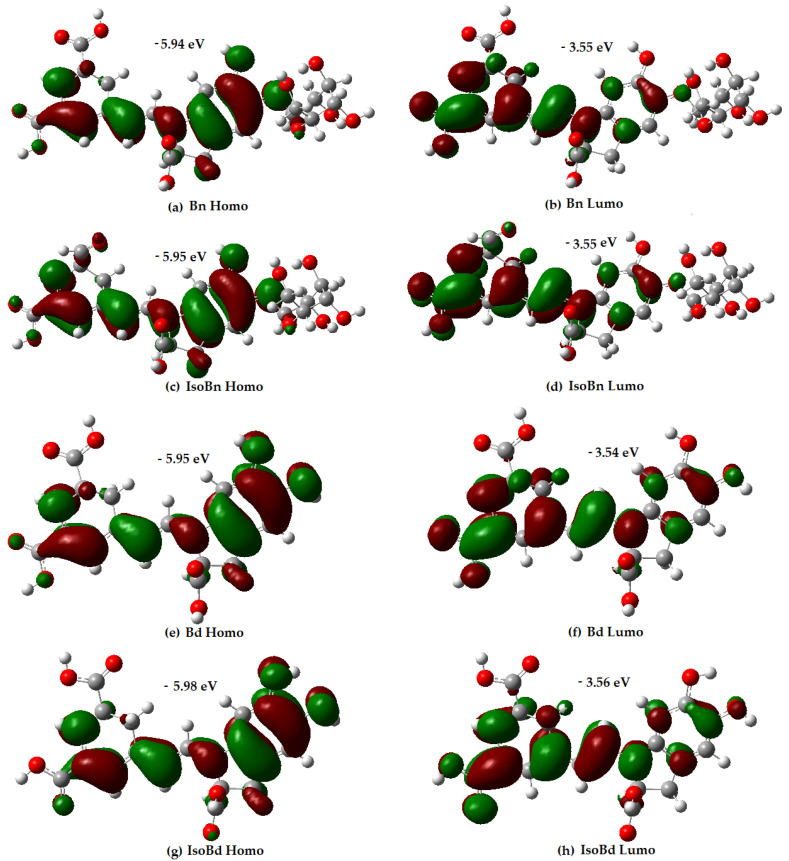
Energy and distribution of HOMO and LUMO for Bn, IsoBn, Bd, IsoBd.

The optimized Bn and C15 epimer (IsoBn), Bd and C15 epimer (IsoBd) structures feature three structural hydrogens that could be released from the molecule, one between the two hydroxyl groups attached to C5 and C6. The other three are formed: two by the oxygen atom of each carboxyl group at C2, C15, and C17 and the third with the hydrogen-bonded to N16. The carboxyl group of the Bd and Bn has an equatorial position, and the epimers in the carboxyl group have an axial position (Supplementary Information, Figure S1). After preparing the molecules, according to the required calculations, which includes O-H (or NH) group in the compound possesses the extractable or directed hydrogen for the radical attack, they were optimized to obtain the lowest energy structures for BnC2, BnC6, BnC15, BnC17, BdN16, BdC2, BdC5, BdC6, BdC15, BdC17, and BdN16, (see Figure 1C and Figure S1), in same way for IsoBn and IsoBd. The antioxidant capacity of these compounds is highly affected by the pH of the medium, but in aqueous solutions, they are stable in a pH range of 3.5 to 7 [16,38,51,52]. To explain the antioxidant activity, we calculated the descriptors BDE, IP, PDE, PA, and ETE on each compound; see Table 1.

The mechanism of scavenging free radicals can predict from the values of the descriptors of the O-H (or N-H) groups in the compounds. By comparing these descriptor values (the PA value is lower than for the rest of the descriptors), the most thermodynamically probable reaction in a polar medium such as water is sequential electron transfer with loss of protons (SPLET). For phenolic antioxidants, experimental and theoretical studies also confirmed the importance of the SPLET mechanism in polar solvents [53].

According to reported data, the carboxyl group is the first in loses the hydrogen, then the hydroxyl group, and finally the N16 group [16,52]. Other authors reported the computation of proton affinity in an aqueous medium. They determined that the ascending order of deprotonation of the Bn and Bd molecules is C17COOH (C17), COOHC15 (C15), and COOHC2 (C2) for Bn and Bd. The PA of the first group with respect to the second and third for Bn is 5.4 Kcal/mol and 9.6 Kcal/mol respectively; for Bd is 5.3 Kcal/mol and 6.8 Kcal/mol, respectively [54]. In a vacuum, the order of deprotonation is C17, C2, and C15 for Bn and Bd [55]. The C17COOH group loses the first hydrogen before all compounds, followed by the hydrogen belonging to the C15COOH (C15) group, and finally the hydrogen corresponding to the C2COOH (C2) group. This is true for most of the compounds except for IsoBn that the value of PA is slightly lower for the C2 group than groups C15, according to the results cited. Comparing the PA values of the C17 group with C15 for all molecules, the range is 2.1 to 3.6 Kcal/mol, and C2 is 2.1 to 3.8 Kcal/mol. The differences between the results obtained and those previously reported are of the same order [54,55]. The lowest PA value corresponds to the hydrogen of the COOH group (C17) attached to the non-chiral carbon of the Bd isomer, followed by the nonchiral group of the Bn isomer. The PA value in kcal/mol of the compounds increases in the following order: IsoBd < IsoBn < Bn = Bd, this indicates that the antioxidant capacity seems to be better at C17 of the Bd epimer. The antioxidant capacity of these compounds includes the next step of the mechanism, related to the descriptor ETE (Equation (2)). The compounds’ ETE value in kcal/mol increases in the following order: Bn < IsoBn < Bd < IsoBd. The fact that IsoBd loses hydrogen easier than the rest and at the same time corresponds to the highest value of the ETE descriptor could imply that the anion form is long-living, which would allow various transformations, thus being able to lose antioxidant capacity [32]. However, in general terms, the difference between the ETE values and all the descriptors is not very wide, and as discussed above, the most favorable antioxidant mechanism in water is the SPLET mechanism for all the compounds studied, IsoBd is the first compound to loss of hydrogen.

The compounds’ IP descriptor in kcal/mol increases as follows: Bn < IsoBn < IsoBd < Bd, implying that Bn possesses the best electron-donating ability [34].

The antioxidant capacity can be evaluated by different in vitro assays, either HAT, SEP assays, or a mixture of both. It depends on the chemical reaction considered and the structure under study [56,57].

Structurally, betalains are immonium derivatives of betalamic acid that contain an aromatic amino compound which can stabilize radicals. This stabilization is tightly linked to betalain’s electron donation ability.

As evidenced by Gandía–Herrero, et al. [51], the antioxidant activity of betalain molecules depends on their chemical structure and can be determined via Mixed-Mode Assays Trolox equivalent antiradical capacity (TEAC) value of betalains without aromatic resonance, charge, or hydroxy groups is 2.4 ± 0.1 units. This value is about 1.8 ± 0.1 units for betalains with charge and no aromatic resonance and increases to 2.8 ± 0.4 units for compounds carrying an aromatic ring. The TEAC value reaches 4.1 ± 0.3 units for betalains having a six-membered benzene ring fused to a five-membered nitrogen containing ring, forming an indoline group. The presence of a charged amino group ion seems to decrease the antiradical activity. Interestingly, no effect related to the carboxylation of these pigments was noted [51]. Specifically the Bn has reached a TEAC of 4.7 ± 0.3 [58].

More recent information presents the assessment of the antioxidant capacity of Bn through four assays, the HAT type: ORAC (10.52), the SET type: FRAP (11.82) and Mixed-Mode Assays are: TEAC (15.35) and DPPH (25.42) [59].

### 3.2. Frontiers Molecular Orbitals (HOMO/LUMO)

HOMO represents the ability to donate electrons, and LUMO the ability to accept electrons. The distribution of the Frontiers molecular orbitals (HOMO/LUMO) in a molecule is related to its antioxidant capacity, as it allows prediction of the most likely sites at which a compound can be affected by free radicals [34].

Analysis of the orbital distribution in Figure 1 shows that the HOMO and LUMO compositions of the compounds exhibit not very marked differences, and they are localized over almost the entire molecule, without any contribution of the glycoside motifs.

The distribution electron density of the LUMO for Bn and its C15-epimer is in higher proportion in the C17COOH group and the dihydropyridine ring concerning the aromatic ring. Regarding the HOMO compositions, the C17COOH group presents delocalization; the aromatic ring shows a slightly different delocalization than the dihydropyridine ring. The results express that the C17COOH group and the dihydropyridine ring are the most likely sites for radical attack for all molecules. The most probable sites for deprotonation are C17, C15, and C2 in all compounds [60]. Additionally, molecules with higher energy HOMO orbital have a stronger electron-donating ability [34]. In Figure 1, Bn has the highest HOMO energy (−5.94 eV), followed by IsoBn and Bd (−5.95 eV), lastly, IsoBd (−5.98 eV). It shows that Bn has the strongest electron-donating ability among the compounds studied, corresponding to the obtained IP value.

### 3.3. IR Analysis

Figure S4 of the Supplementary Material shows the evolution of parent molecules and C15-epimers, corresponding to a rotation. The magnitude of the interactions present is explained exclusively by kinetic effects. That is why the Bn and its Iso have glucose. It is a more significant vibrational load. There is a region of vibration of the glucose in the range of 905.10 cm${}^{-1}$ to 849 cm${}^{-1}$. The first hydrogen that is lost in the reaction is affected by the number of additional bonds that the molecule has; that is to say, deprotonation is facilitated to the extent that the molecule has a greater number of atoms, which is correlated in lower energy in IR’s intensities with the following order Bn < IsoBn < Bd < IsoBd.

**Figure 2:**
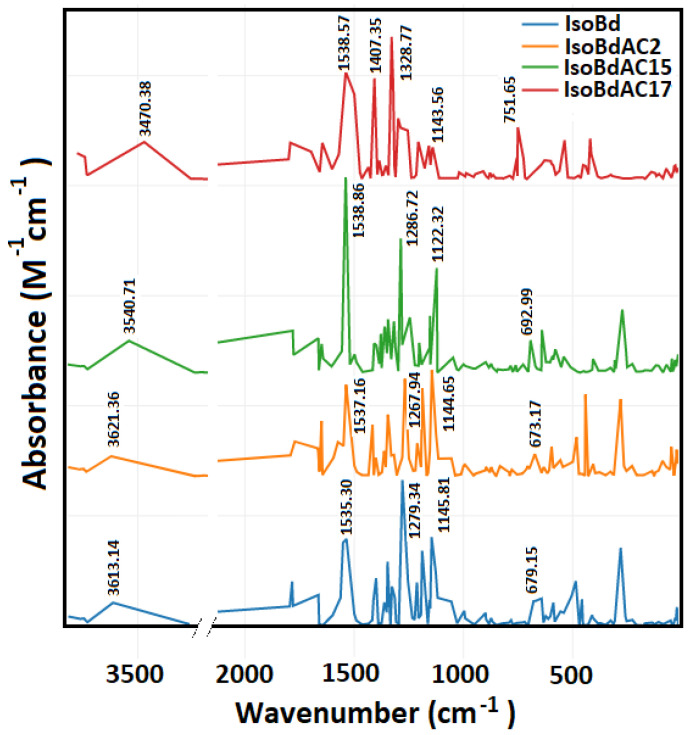
Parent Isobetanidin (IsoBd) and their anios IsoBdAC2, IsoBdAC15, and IsoBdAC17.

In Figure 2, we can see the IR spectrum of the anions C2COOH, C15COOH, and C17COOH of IsoBd. Intense peaks at 1538.57 (*b*,*s*) cm^−1^, 1538.86 (*s*) cm^−1^, and 1537.16 (s) cm^−1^ belong to C=C stretch mode for C2COOH, C15COOH, and C17COOH complex, this behavior at phenyl compounds with pair of bands only for the parent IsoBd and anion C17COOH has the origin of the degenerate pair of fundamentals in benzene. The overall displacement in benzene results in a change in dipole moment [61]. At 1407.35 (*s*) cm^−1^, the C17COOH presents δ CO stretch mode more attenuated in other anions. The whole of more intense peaks also can see at 1328.77 (*s*) cm^−1^, 1286.72 (*s*) cm^−1^ and, 1267.94 (*s*) cm^−1^ for C17COOH, C15COOH, and C2COOH anions, respectively, belong to ($\nu_{as}$ C−N) asymmetric stretching. The bands observed at 1143.56 (*w*) cm^−1^, 1122.32 (*m*) cm^−1^, and 1144.65 (*m*) cm^−1^ for C17COOH, C15COOH, and C2COOH complex could be attributed to the C=C stretch mode; at C17COOH anion, their intensity is attenuated that could be indicated loss energies vibrations and a reconfiguration of the kinetic. Furthermore, it observed a bathochromic shift for the anion C17COOH in all absorptions frequencies implying an increase in velocity the loss of hydrogen [16,62,63]. This behavior correlated the same outcomes with the Shape Theory and DFT simulations. Out-of-plane (ω C−H) bend (aromatic ring), 751 (*b*,*m*) cm^−1^ for C17COOH. The fingerprint area near 1500 cm^−1^ is a good characterization of the deprotonated (loss hydrogen) change. According to the literature, the range of 1700–1400 cm^−1^ contains the series of maximum characteristics of COO- and the amine groups [64]. The amide N atoms have a broadband region of 3700 to 3200 cm^−1^ (νNH); with an appropriate deconvolution, it is possible to find peaks corresponding to the coordination of deprotonated peptides of complex or ligands with these characteristics [64,65]

### 3.4. Shape Space Distances

The molecules studied in the present work showed marked variations the antioxidant capacity. We found that the hydrogen is lose firstly in the C17COOH group in all the studied compounds. Similar researches for Bd, Bn, and IsoBd show that the first deprotonation occurs at C17COOH, C2COOH and C15COOH [38] and in [54] occurs at C17COOH, C15COOH and C2COOH. The last results agree with the present study for the case of Bn and Bd. However, an explicit relation with the geometry of the molecules could be found and a parametrization of chemistry descriptor could be related with the invariant shape theory analysis. Table 2 shows that this is possible. The values of the PA and RD (global and by subspaces) are described for all of the studied compounds and anions. We note that the global DR preserves the order of the PA descriptor. Concerning the PA, the order between each compound and its respective C15-epimer is conserved, indicating that the most stable deprotonated molecule is also the closest to the parent compound.

When the RDs are compared among clusters of the same molecule, a similar conclusion is obtain. The RDs by subspaces define stages of verification, given that the partial distance inherits the global distance of the complete molecule, which is expect harmonically balanced and optimized in all the group elements.

This surprising result complements the usual analysis defined by the physical and chemical descriptors. The intrinsic Riemannian geometric properties of the optimized molecule are related with the first deprotonated group and the most stable molecule.

In this case, the most consistent RD (global and partial) reveals that C17COOH is the first deprotonated group in all the four compounds. These molecules exhibit the lowest proton affinity and at the same time show the lowest RD of that anion to the corresponding parent molecule.

### 3.5. New Shape Molecular Docking

This section aims to visualize the possible interactions of the selected compounds in complex with the XO enzyme and to elucidate the potential mechanism of action of this group of molecules in preventing ROS formation. The literature widely reported the antioxidant capacity of betalains [16,39,51,52]. Additionally, other studies [12,13,66] showed that in a reducing half-reaction, the substrate is hydroxylated oxidatively at the molybdenum (Mo) center, whose deprotonation can be facilitated by a conserved glutamate residue (Glu1261 in the bovine enzyme). Xanthine is formed from the hydroxylation of hypoxanthine at C-2 (see Figure S5) and is then converted to uric acid by hydroxylation at C-8 (see Figure S5), which involves the generation of ROS. If the XO enzyme is inhibited, it could be a way to treat or prevent diseases related to uric acid accumulation [67]. The XO inhibitor considered as a reference in this study is the drug Febuxostat, which is not affected by the redox status of the enzyme and the interaction with XO. It is also very potent in antioxidant activity and is very well coupled to the XO pocket that obstructs substrate binding [14]. We explore the probable interactions of the union of the compounds with XO via molecular docking in the binding sites of the protein made up of the amino acid residues within and around the binding pockets of the hypoxanthine and molybdopterin cofactor, framed within the C chain and delimited by the grid box defined to enclose the active site according to the methodology. This complex with XO yielded binding energy (ΔG) of −9.0 kcal/mol. The reference showed hydrogen bonding interactions with the amino acids Asn768, Arg880, and Thr1010. In addition, it presented hydrophobic interaction with amino acids Phe1009, Phe1013, Leu873, Val1011, Leu1014, and Ala1079. Other hydrophobic bonds shown were with Ala1079 and Phe914, respectively. The above agrees with results found in the literature [15,33,67].

**Figure 3:**
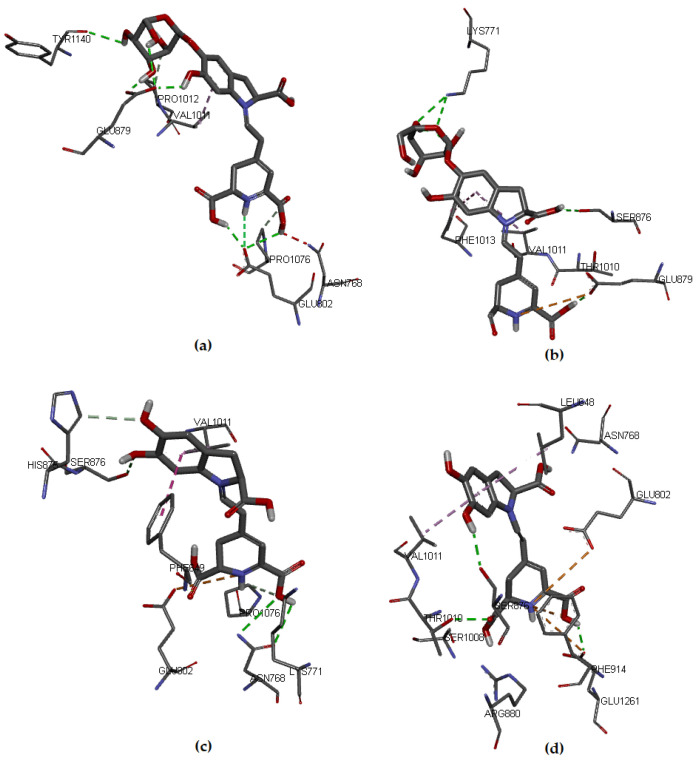
Molecular models of binding of compounds to active site of xanthine oxidase. A three-dimensional model (Docking): (**a**) Bn, (**b**) IsoBn, (**c**) Bd, and (**d**) IsoBd.

Regarding the studied molecules, Bn, Bd, and their respective C15-epimers, the docking results showed that IsoBd and Bn could be more active since their a lower ΔG value, which corresponds to −8.0 kcal/mol and −7.5 kcal/mol, respectively, while IsoBn reached a value of −7.0 kcal/mol and Bd −6.9 kcal/mol. These molecules formed stable complexes with XO. The interaction of the compounds with the target was favorable through nonbond interactions, primarily by hydrogen bonding and in smaller proportion by hydrophobic and electrostatic interactions (Figure 3). For example, for Bn, a hydrogen bond (2.136 Å) was formed between the hydrogen atom of the carboxylic acid of the non-chiral carbon of Bn and the oxygen atom of the residue Glu802. It occurs similarly between the oxygen atom of the same residue with the hydrogen atom of the carboxylic acid of the chiral carbon of Bn (2.934 Å). We observe Hydrogen bonding type interactions between hydrogens of the glucose of Bn and the Glu879 residue with distances of 2.667 Åand 1.971 Å, respectively.

There is an interaction between the hydrogen corresponding to the hydrogen of the ligand and the oxygen of Glu802 (2.934 Å). In Figure 3a, we present the hydrophobic interaction between the Bn’s aromatic ring and Val1011, distance in Åat 4.357. In IsoBn (see Figure 3b), hydrogen bonds can be observed with residues Lys771 (2.855 Å), Ser876 (2.810 Å), and Glu879 (1.877 Å), in addition to an electrostatic-type bond with residue Glu879 (5.323 Å). Furthermore, there are hydrophobic interactions with Phe1013 (5.292 Å) and Val1011(5.466 Å). Figure 3c shows that the interactions of Bd with the protein pocket are of hydrogen bonding type; the amino acids involved in such interactions are His875 (2.316 Å), Glu879 (2.403 Å), Ser876 (3.711 Å), electrostatic type with residue Glu879 (3.326 Å) and hydrophobic interactions with residues Leu648 (4.848 Å) and Val1011 (4.534 Å). IsoBd (see Figure 3d) exhibited electrostatic interactions with amino acids: Glu802 (5.305 Å), Phe914 (3.431 Å), and Glu1261 (5.211 Å), hydrogen bonding type with Thr1010 (2.510 Å), Glu1261 (2.147 Å) and Ser876 (2.267 Å) and hydrophobic interactions were present with residues Leu648 (4.834 Å) and Val1011 (4.399 Å).

According to the results, Bn and IsoBd interact favorably with the active site of XO through the formation of hydrogen bonds with the C15-hydroxyl group and the C17-hydroxyl group in the case of Bn and an electrostatic interaction for the possibility of IsoBd generating inhibition in the Glu802 residue. We could say that IsoBd can behave as a competitive inhibitor of the XO enzyme since it interacts with Glu1261 in the active site, which is responsible for the deprotonation of the Mo-OH prosthetic group in the enzyme. After deprotonation, the nucleophilic attack on the substrate occurs, promoting the oxidation reaction, generating ROS [14]. In general, the interactions of the this compound with the protein pocket showed favorable interactions, allowing the inhibition of the protein. This situation is critical at the cellular level since oxygen-based radicals are associated with a series of events, including the activation of nicotinamide adenine dinucleotide phosphate oxidase (NADPH), cytochrome catalyzed linoleate peroxidation, xanthine oxidase (XO), which is a form of xanthine oxidoreductase that generates reactive oxygen species, such as radicals superoxide and hydrogen peroxide [13,66].

The values of the binding affinities of the compounds are in a not very wide range (−6.9–−8.0 Kcal/mol, there is not much difference and above Febuxostat. Furthermore, these compounds interact with amino acids Val1011, Phe914, Phe1013, Glu802, Glu1261, or Thr1010, amino acids involved in interactions with the reference ligand Febuxostat and with the control ligand Hypoxanthine reported in the literature [15,33]. These results imply that the molecules studied bind to XO at the active site of the cofactor molybdopterin, allowing the interruption of the catalytic pathway for the activation of the enzymatic action of XO which is to generate the superoxide radical anion.

**Figure 4:**
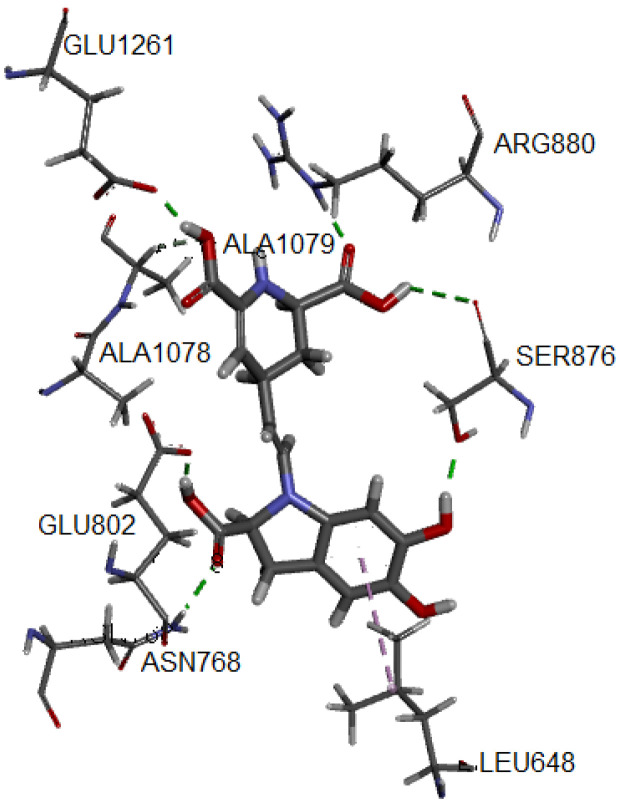
Hydrogen bonds and alkyl interactions of IsoBd with XO obtained from MD study.

One of the most common measures to evaluate the condition of the complex along the trajectory is the quantification of the structural formation compared to the initial reference point. This evaluation is performed by the root mean square deviation (RMSD) of the main structure of the protein with the studied ligand, compared to the reference structure. In Figure S6 of the Supplementary Material, the complex formed with IsoBd has high stability during the simulation; the setting does not vary significantly, as in this case, there is an ascent of the RMSD until reaching a point where the values fluctuate around of 0.07 and 0.12 nm of RMSD. After of 10 ns, the structure remains within the parameter that considers the system to be in equilibrium; the complex did not lose its structure during the simulation. We used the VMD graphic viewer to obtain the trajectory (frame) corresponding to the most stable configuration to analyze this complex better. Figure 4 presents the interactions between the IsoBd ligand and XO.

The hydrogen bond correspond to the residues Glu1261 (1.741 Å), Arg880, (1.696 Å), Ala1079 (2.364 Å), Glu802 (1.592 Å), Ser876 (3.055 Å) and Asn768 (2.045 Å). On the other hand, the complex presents an interaction of alkyl type marked by a violet segmented line with the Ala1078 residue. Both the enhanced stability and antiradical of betalains may be explained by the formation of hydrogen bonding, such as an adjacent hydroxyl or amino group and the betalains contain more than one phenolic hydroxy group. The polar nature of the O–H bond of the phenol group results in the formation of hydrogen bonds with other phenol molecules or other H-bonding systems and implies a high solubility in aqueous media. The radical quenching activity of betalain pigments is basically supported by the “intrinsic activity” shared between the imino and the tetrahydropyridine groups [68].

Regarding the implementation of the docking method based on shape theory through the definition of an initial ligand arranged according to the maximum variability along the *x*-axis and rotated to maintain the remaining variabilities in the *y*- and *z*-axes, it observed that the results compete in quality with those obtained by the expert algorithm of the molecular docking software as Autodock Vina. Specifically, one of the method’s main findings consisted of improving or equaling the bond affinities obtained by the software, as mentioned earlier, with the additional advantage that this method is entirely geometric. In addition, we noted that all 10 ligands proposed for each molecule yielded ΔG values consistent in most cases with those obtained by the expert software (see Table 3).

This method modifies the ligand coordinates, by using its Riemannian geometric properties. The protein (pocket) is not perturbed because it is fixed by an expert. Finally, the Autodock Vina software optimizes the protein-ligand complex according to the corresponding chemical interactions.

**Figure 5:**
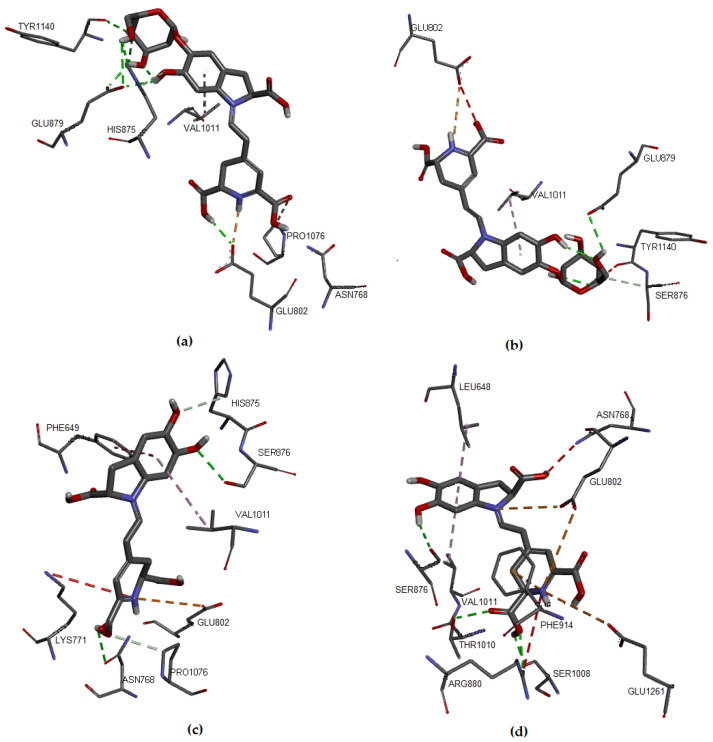
Molecular models of binding of compounds to active site of xanthine oxidase. A three-dimensional model based on shape theory (**a**) Bn, (**b**) IsoBn, (**c**) Bd, and (**d**) IsoBd.

Figure 5 shows the interactions in the protein-ligand complex, where the ligand coordinates were computed by our algorithm.

The results obtained for binding affinities were very similar to those previously obtained from the Euclidean coordinates of the protein-ligand complex (Table 3), showing that IsoBd and Bn possess the lowest ΔG value, followed by Bd and IsoBn. The interactions with the amino acids of the protein pocket are listed in Table 4, Table 5, Table 6 and Table 7. We highlight that IsoBd presented a more significant number of interactions, which is in agreement with other investigations about antioxidant capacity [11,13,33].

The range of values of affinities of all compounds biding is between −6.2 kcal/mol −8.1 kcal/mol and exceeds the binding affinity of the reference ligand Febusxostat (−9.1 kcal/mol) obtained by applying the same method. Regarding the reference interactions, their residues are Arg880, Thr1010, Phe914, Ala1079, Ala1078, Leu873, Val1011, and Leu1014, which are in the same line as those reported in other works [15,33].

## 4. Conclusions

We analyze the antioxidant activities of some betacyanins (Bn, IsoBn, Bd, and IsoBd) via Shape Theory, Molecular Dynamics, Density Functional Theory, and Infrared Spectroscopy. The antioxidant activity of the compounds considered in this study were analyzed at the B3LYP level of theory and with the basis set 6-31+G(d,p). We find it important to include spin-contamination analysis via UB3LYP, which is often omitted in numerous applications of DFT to catalysis. The values’ descriptors BDE, PA, ETE, IP, and PDE evidenced water’s most likely reaction mechanism. Furthermore, the calculation showed that the SPLET mechanism is more likely than the HAT and SET-PT mechanisms since the PA values are lower than IP and BDE. Additionally, the group that loss the hydrogen first is C17COOH. We constructed our own algorithms based on shape theory. The results conclude that the order of the RDs is consistent with the order of the PA values. In this case, the lowest PA and RD values indicates the optimal mechanism of the antioxidant activity for studied molecules. The assessments of algorithms also establish a consistent results between the global and partial RD for groups of elements. This fact provides a number of checking rule for choosing the best mechanism of antioxidant capacity. The method of shape theory also defines a routine for molecular docking based on desirable invariant properties of the Riemmanian geometric. The new molecular docking studies showed that most of the compounds presented interactions with XO. In addition, molecular dynamics for IsoBd compound showed interactions with a higher number of amino acids belonging to the protein pocket. A similar behavior was found for the Febuxotat, the antioxidant drug used as reference.

About the molecular dynamic, the study of the free radical scavenger via MD is explained by the formation of hydrogen bonding, preserved during the simulation. This characteristic is in accordance with previous results of the reference molecule.

The new method of molecular docking for the ligand shows some correspondence with the results of the molecular docking carried out with the expert software Autodock Vina. As robust strategy, a method that integrates the protein coordinates will be propose in a future work by the authors.

We found in IR spectra from IsoBd a correlation between a bathochromic shift for the anion C17COOH in all absorptions frequencies alone an increase in velocity the loss of hydrogen. We proved the chemical stability of the molecules in regions of the amine at 1700 to 1300 cm^−1^ and 3700 to 3200 cm^−1^ that corresponding to the coordination of deprotonated peptides of complex or ligands.

In a future work, we could explore the link of the neural networks under nonlinear models with predictors of preshapes, studied in the Riemannian hyper-sphere. This translation into the well know language of artificial intelligence can include predictors and responses involving the geometric invariances of the Riemannian Shape Theory. 

## Figures and Tables

**Table 1:** Calculated BDE, PA, ETE, IP, and PDE values for compounds studied. Data in kcal/mol, at B3LYP/6-31+G(d,p) level of theory in water.

Compound	Bond	HAT	SPLET		SET-PT	
		BDE	PA	ETE	IP	PDE
Bn	C2	84.8	15.0	69.9	74.1	10.7
	C15	84.1	14.2	69.9		10.0
	C17	79.6	**11.6**	68.1		5.5
	N16	83.7	23.3	60.4		9.6
	C6	75.4	26.2	49.2		1.3
IsoBn	C2	85.0	15.0	70.0	74.2	10.8
	C15	84.6	15.2	69.4		10.4
	C17	79.6	**11.4**	68.2		5.4
	N16	84.0	23.7	60.2		9.8
	C6	75.4	26.2	49.2		1.2
Bd	C2	85.3	14.9	70.4	75.0	10.3
	C15	84.8	14.2	70.6		5.2
	C17	80.2	**11.6**	68.6		9.8
	N16	83.8	23.2	60.6		8.8
	C6	73.3	22.9	50.4		−1.7
	C5	75.4	25.8	49.6		0.4
IsoBd	C2	85.1	14.3	70.8	74.8	10.3
	C15	83.9	12.9	71.0		9.0
	C17	80.0	**10.8**	69.2		5.2
	N16	81.7	20.8	60.9		6.9
	C6	77.5	26.7	50.7		2.7
	C5	70.6	17.6	53.0		−4.3

**Table 2:** RD between study compounds and their respective anions and PA values.

Compound	PA	DR	DR by Subspaces		
			O	C	H
BnAC15	14.2	0.013	0.021	0.005	0.006
BnAC17	11.6	0.010	0.015	0.006	0.009
BnAC2	15.0	0.011	0.014	0.007	0.009
BnAC6	26.2	0.013	0.011	0.011	0.014
BnAN16	23.3	0.036	0.059	0.014	0.022
IsoBnAC15	15.2	0.011	0.012	0.007	0.010
IsoBnAC17	11.4	0.008	0.008	0.008	0.008
IsoBnAC2	15.0	0.011	0.012	0.010	0.012
IsoBnAC6	26.2	0.011	0.010	0.008	0.013
IsoBnAN16	23.7	0.021	0.031	0.014	0.012
BdAC15	14.2	0.013	0.021	0.004	0.006
BdAC17	11.6	0.008	0.008	0.007	0.009
BdAC2	14.9	0.009	0.008	0.008	0.008
BdAC5	25.8	0.021	0.018	0.016	0.023
BdAC6	22.9	0.013	0.010	0.010	0.014
BdAN16	23.2	0.034	0.054	0.013	0.022
IsoBdAC15	12.9	0.012	0.013	0.009	0.012
IsoBdAC17	10.8	0.007	0.005	0.008	0.007
IsoBdAC2	14.3	0.013	0.023	0.005	0.005
IsoBdAC5	17.6	0.070	0.035	0.013	0.098
IsoBdAC6	26.7	0.070	0.031	0.010	0.099
IsoBdAN16	20.8	0.061	0.099	0.015	0.046

**Table 3:** ΔG of compounds under study and reference considering geometry of ligands founded on shape theory and one calculated by expert software.

	ΔG Kcal/mol				
Rotation	Bn	IsoBn	Bd	IsoBd	Reference
					(Febuxostat)
None	−7.5	−6.3	−6.8	−8.1	−7.8
Plane xy 90°	−7.5	−7.0	−6.9	−8.0	−9.1
Plane xy 180°	−7.5	−5.9	−6.9	−8.0	−9.1
Plane xy 270°	−7.5	−5.9	−6.8	−8.1	−9.1
Plane xz 90°	−7.5	−5.9	−6.7	−8.1	−9.1
Plane xz 180°	−7.5	−5.9	−6.9	−8.1	−9.1
Plane xz 270°	−7.5	−6.1	−6.8	−8.1	−9.1
Plane yz 90°	−7.5	−5.9	−6.8	−8.1	−9.1
Plane yz 180°	−7.5	−5.8	−6.8	−7.3	−9.1
Plane yz 270°	−7.5	−6.2	−6.8	−8.1	−9.1
ΔG (Kcal/mol) Autodock Vina	−7.5	−7.0	−6.9	−8.0	−9.0

**Table 4:** Docking results of binding of Bn to active site of xanthine oxidase.

Compound	ΔG (kcal/mol)	D-Bond Å	Type	Amino acids
Bn	−7.5	2.379	Hydrogen Bond	Glu802
		2.163	Hydrogen Bond	Glu802
		2.159	Hydrogen Bond	Glu879
		2.078	Hydrogen Bond	Glu879
		2.439	Hydrogen Bond	Tyr1140
		3.065	Hydrogen Bond	His875
		3.474	Hydrogen Bond	Pro1076
		4.359	Hydrophobic	Val1011

**Table 5:** Docking results of binding of IsoBn to active site of xanthine oxidase.

Compound	ΔG (kcal/mol)	D-Bond Å	Type	Amino Acids
IsoBn	−6.2	4.573	Electrostatic	Glu802
		2.750	Hydrogen Bond	Glu879
		3.698	Hydrogen Bond	Ser1141
		4.963	Hydrophobic	Val1011

**Table 6:** Docking results of binding of Bd to active site of xanthine oxidase.

Compound	ΔG (kcal/mol)	D-Bond Å	Type	Amino Acids
Bd	−6.9	5.008	Electrostatic	Glu802
		2.705	Hydrogen Bond	Ser876
		2.949	Hydrogen Bond	Asn768
		3.508	Hydrogen Bond	His875
		3.407	Hydrogen Bond	Pro1076
		4.777	Hydrophobic	Phe649
		5.086	Hydrophobic	Val1011

**Table 7:** Docking results of binding of IsoBd to active site of xanthine oxidase.

Compound	ΔG (kcal/mol)	D-Bond Å	Type	Amino Acids
IsoBd	−8.1	5.246	Electrostatic	Glu802
		5.192	Electrostatic	Glu1261
		2.515	Hydrogen Bond	Arg880
		2.536	Hydrogen Bond	Thr1010
		2.729	Hydrogen Bond	Ser876
		3.449	Electrostatic	Phe914
		4.688	Hydrophobic	Leu648
		4.562	Hydrophobic	Val1011

## Data Availability

Ramirez Velásquez, Iliana (2021), “Antioxidant activities of betanin, betanidin and respective C15-epimers via DFT methods, shape theory and molecular docking”, Mendeley Data, V1, doi:10.17632/vvvxj44s9y.1.

## Supplementary Materials

The following supporting information can be downloaded at: https://www.mdpi.com/article/10.3390/molecules27062003/s1. Figure S1. Basic structure of Betacyanins. (**a**): Betalamic Acid, (**b**): cyclo-DOPA, (**c**): Betacyanins R = Glc (Betanin), R = H (Betanidin). Figure S2. Epimer at C15 of Betanin and Betanidine. Figure S3. Optimized structures obtained for Betanin (Bn), Isobetanín (IsoBn), Betanidin (Bd) and Isobetanidin (IsoBd). Method used/IEFPCM/ B3LYP /6-31+G(d,p). Solvent: water. Coordinates of the geometry optimized with IEFPCM/ B3LYP /6-31+G(d,p). Solvent: water. Geometry of IsoBd and anions. Figure S4. Parent molecules for Betanine (Bn), Isobetanina (IsoBn), Betanidine (Bd), and Isobetanidine (IsoBd). Figure S5. Chemical structures and numbering scheme of xanthine and hypoxanthine. Table S1. IR data for Bn, IsoBn, Bd, and IsoBd. Figure S6. RMSD of the protein backbone during the simulation of DM trajectories.

## Abbreviations

The following abbreviations are used in this manuscript:

Affine	is a geometric transformation that preserves lines and parallelism
BDE	bond dissociation enthalpy
Bd	betanidin
Bn	betanin
B3LYP	Becke, 3-parameter, Lee–Yang–Parr
DFT	Density Functional Theory
DNA	Deoxyribonucleic acid
DPPH	2,2-Diphenyl-1-Picrylhydrazyl Assay
ETE	the electron transfer enthalpy
FAD	flavin adenine dinucleotid
FRAP	Ferric Reducing Antioxidant Power Assay
HAT	hydrogen atom transfer mechanism
HOMO	highest occupied molecular orbital
IC50	The maximum mean inhibitory concentration
IEFPCM	Integral-Equation-Formalism Polarizable Continuum Model
IP	ionization potential
IR	infrared spectra
IsoBd	isobetanidin
IsoBn	isobetanin
LUMO	lowest unoccupied molecular orbital
MD	Molecular Dynamic
Mo-pt	Molybdopterin
NADPH	nicotinamide adenine dinucleotide dinucleotide phosphate oxidase
ORAC	Oxygen Radical Absorbance Capacity Assay
PA	proton affinity
PDE	proton dissociation enthalpy
QR	is a A = QR of an orthogonal matrix Q and an upper triangular matrix R
RD	Riemann distance

RNS	Reactive Nitrogen Species
ROS	Reactive Oxygen Species
SET-PT	sequential electron proton transfer mechanism
SLEPT	sequential proton-loss electron transfer mechanism
SVD	singular value decomposition
TEAC	the Trolox Equivalent Antioxidant Capacity
VMD	visual molecular dynamics
XO	Xanthine oxidse
