# Supplementary material for: Mechanism of Antioxidant Activity of Betanin, Betanidin and Respective C15-Epimers via Shape Theory, Molecular Dynamics, Density Functional Theory and Infrared Spectroscopy
Source: Molecules. 2022 Mar 21;27(6):2003. doi: 10.3390/molecules27062003 (PMC8954076; doi:10.3390/molecules27062003)
Supplement: Supplementary file 1 [file molecules-27-02003-s001.zip › Supplementary material.pdf]

Mechanism of Antioxidant Activity of Betanin, Betanidin and respective C15-Epimers via Shape Theory, Molecular Docking, Molecular Dynamic and DFT Methods including IR

Ramírez Iliana[1,2], Vélez Ederley[2], Bedoya Alvaro[2], Caro Francisco[2]

[1]Instituto Tecnológico Metropolitano, Facultad de Ciencias Exactas y Aplicadas, Calle 73 No. 76A – 354. Corresponding author: ilianaramirez@itm.edu.co
[2]Universidad de Medellín, Facultad de Ciencias Básicas, Cra. 87 #30-65, Medellín, Antioquia, Colombia.

Abtsract: Betanin and betanidin are compounds that have extensive interest; they are effective free radical scavengers. The present work aims to elucidate the differences between the mechanism of the antioxidant activity of betanin, betanidin, and their respective C15-epimers. This Shape Theory is used for this objective to establish comparisons between the molecules' geometries and, with those, to determine parallelisms with the descriptors: BDE, PA, ETE IP, PDE, and with the infrared spectra (IR) obtained from the molecule simulations. Furthermore, the molecules were optimized using the B3LYP/6-31+G(d,p) protocol. Finally, the molecular docking technique analyzes the antioxidant activity of the compounds in complex with the therapeutic target xanthine oxidase (XO), based on a new proposal for the geometrical arrangement of the ligand atoms in the framework of Shape Theory. The results obtained indicate that the SPLET mechanism is the most favorable in all the molecules studied and that the first group that loses the hydrogen atom in the four molecules is the C17COOH, presenting less PA the isobetanidin. Furthermore, regarding the molecular docking, the interactions of these compounds with the target were favorable, standing out to a greater extent the interactions of isobetanidin with XO, which were analyzed after applying molecular dynamics.

Keywords: Antioxidant mechanisms; DFT calculations; Shape theory; Molecular docking.

SUPPORTING INFORMATION

Table of contents

Figure S1. Basic structure of Betacyanins. a): Betalamic Acid, b): cyclo-DOPA, c): Betacyanins R = Glc (Betanin), R = H (Betanidin).

Figure S2. Epimer at C15 of Betanin and Betanidine.

Figure S3. Optimized structures obtained for Betanin (Bn), Isobetanín (IsoBn), Betanidin (Bd) and Isobetanidin (IsoBd). Method used/IEFPCM/ B3LYP /6-31+G(d,p). Solvent: water

Coordinates of the geometry optimized with IEFPCM/ B3LYP /6-31+G(d,p). Solvent: water

Geometry of IsoBd and anions

Figure S4. Parent molecules for Betanine (Bn), Isobetanina (IsoBn), Betanidine (Bd), and Isobetanidine (IsoBd).

Table S1. IR data for Bn, IsoBn, Bd, and IsoBd

Figure S5. Chemical structures and numbering scheme of xanthine and hypoxanthine

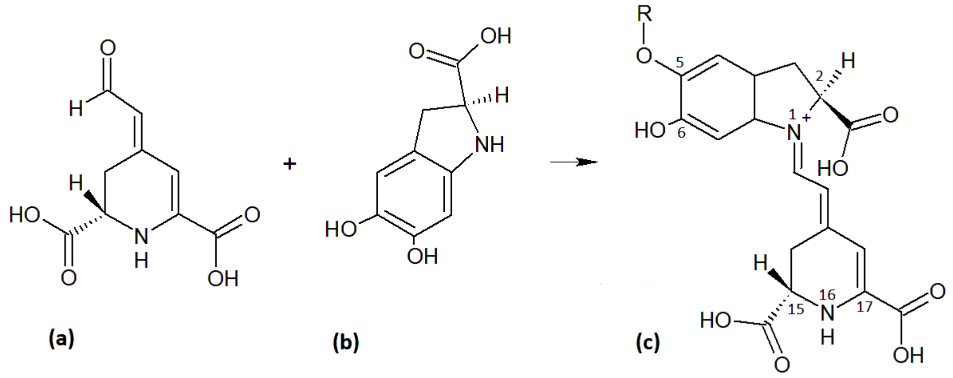

Figure S1. Basic structure of Betacyanins. a): Betalamic Acid, b): cyclo-DOPA, c): Betacyanins R = Glc (Betanin), R = H (Betanidin).

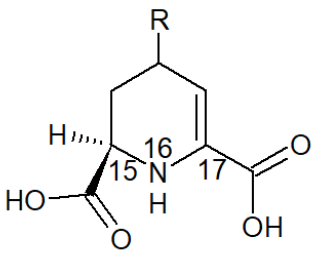

Figure S2. Epimer at C15 of Betanin and Betanidine.

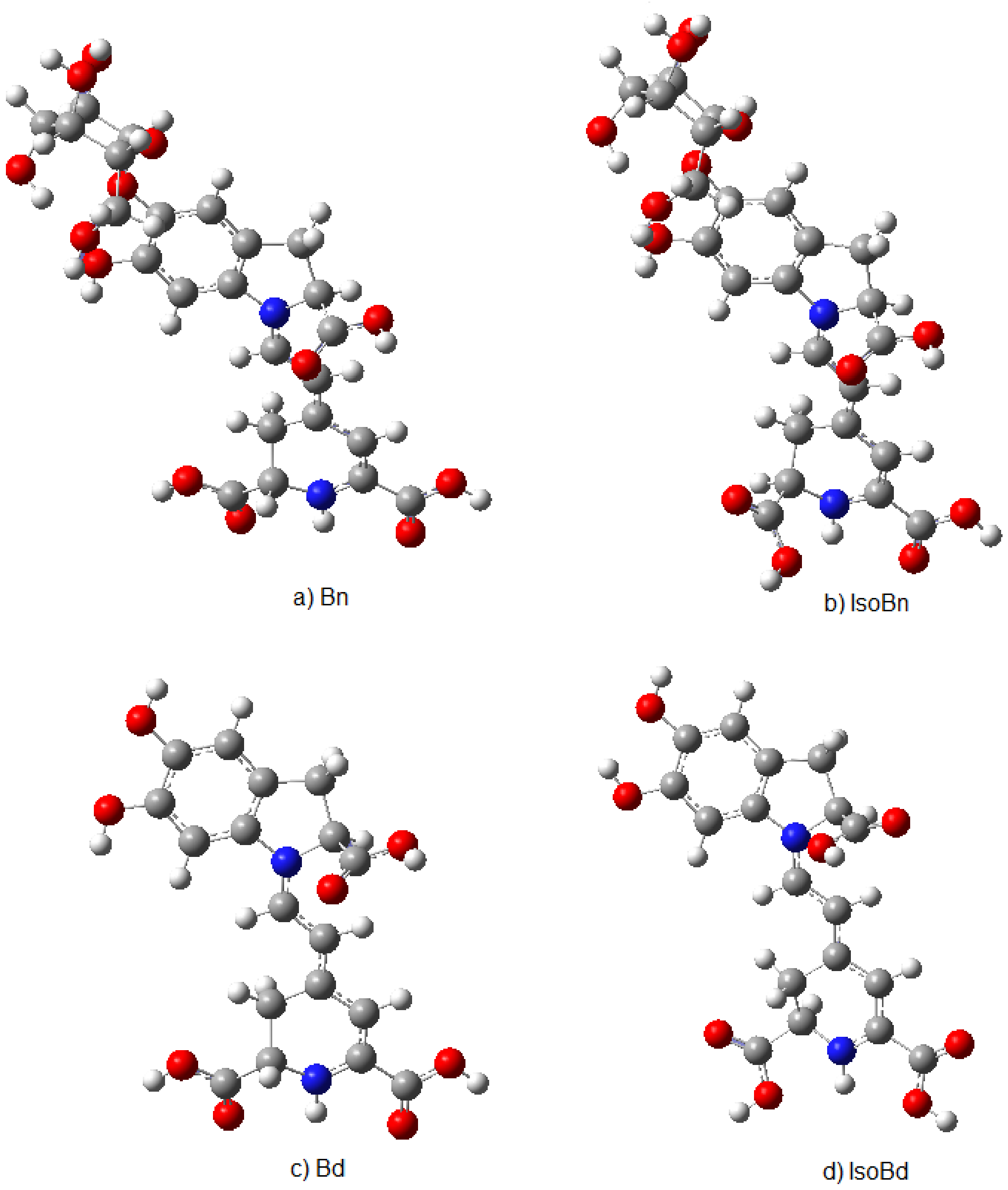

Figure S3. Optimized structures obtained for Betanin (Bn), Isobetanín (IsoBn), Betanidin (Bd) and Isobetanidin (IsoBd). Method used/IEFPCM/ B3LYP /6-31+G(d,p). Solvent: water

Coordinates of the geometry optimized with IEFPCM/ B3LYP /6-31+G(d,p). Solvent: water

We present the coordinates of the geometries optimized of Bn, IsoBn, Bd and IsoBd. Method used/IEFPCM/ B3LYP /6-31+G(d,p). Solvent: Water.

Bn

Electronic Energy (a.u.) = -2018,223551

1 1

C	0.22073100	-0.38684400	-0.34190300
N	-1.06062600	-0.99482600	-0.32283100
C	1.19088900	-1.32832800	-0.66934700
C	0.53699200	0.94941700	-0.08604500
C	-0.91370600	-2.46147000	-0.45272600
C	-2.22517700	-0.35891000	-0.22702800
C	0.55915100	-2.67414600	-0.91963300
C	2.52945800	-0.94939800	-0.75251400
C	1.87251600	1.33220300	-0.18208100
C	-1.17622600	-3.14938300	0.89011900
C	-3.48154700	-0.96778200	-0.19161300
C	2.87684500	0.38531500	-0.51916000
O	2.28841600	2.61172300	0.04089200
O	-1.23172300	-2.59373800	1.96624400
C	-4.68659400	-0.26466800	-0.13813300
O	4.14324400	0.88744400	-0.58508200
C	-4.76324700	1.25192100	-0.18956700
C	-5.91318600	-0.96851600	-0.15390900
C	5.19424600	0.08672400	-1.12800600
C	-6.03342900	1.75993100	0.51470300
C	-7.12062900	-0.28559500	-0.15362800
C	6.36087100	1.03879100	-1.44000100
O	5.55565300	-0.99390000	-0.29895700
N	-7.19977400	1.03707600	0.04242800
C	-6.27811800	3.25408800	0.34779000
C	-8.44649100	-0.95533400	-0.38085300
C	7.13986900	1.45406100	-0.17163500
O	7.22459700	0.41845600	-2.39215700
C	6.36573500	-0.78211000	0.88322200
O	-5.18524900	3.96847900	0.64003800
O	-7.34701400	3.73251600	0.02504200
O	-8.34228600	-2.27290200	-0.57626200
O	-9.49363600	-0.33466600	-0.38627800
C	7.52691000	0.19889700	0.64460800
O	6.45636400	2.42372800	0.60431800
C	5.54180000	-0.54858800	2.14749900
O	8.52083300	-0.55079600	-0.08972100
O	4.97821600	0.76944800	2.20617600
H	-0.21189500	1.68511300	0.18632800
H	-1.62452300	-2.84104700	-1.18882900
H	-2.14426600	0.72033400	-0.18227400
H	0.57976500	-2.93868900	-1.98143100
H	1.04336300	-3.48507600	-0.37036200

H	3.29196700	-1.68349200	-0.97929900
H	-3.55025900	-2.05018800	-0.21556400
H	1.53088600	3.17866100	0.24647700
H	-3.90334400	1.72650000	0.28153400
H	-4.76971100	1.55728700	-1.24443400
H	-5.92313100	-2.04690800	-0.23935500
H	4.84850100	-0.37891900	-2.05589600
H	-5.92015900	1.58322800	1.59759900
H	5.95047400	1.93267000	-1.91573600
H	-8.10696000	1.49228400	0.04308900
H	8.06658900	1.93164800	-0.50799100
H	7.80557100	-0.19405200	-1.90517600
H	6.83037600	-1.76099700	1.03802000
H	-5.39271400	4.91703100	0.55818900
H	-9.23272600	-2.64352700	-0.71223600
H	7.93380900	0.50754900	1.61396000
H	5.78993500	1.97512600	1.16659100
H	4.74139300	-1.29680600	2.17717100
H	6.20471900	-0.71326400	3.00511500
H	9.36667900	-0.08176200	-0.04169800
H	4.74913500	0.97713200	3.12201700
O	-1.31251500	-4.47155100	0.70958200
H	-1.43584900	-4.90120100	1.57536600

IsoBn

Electronic Energy (a.u.) = -2018,222698

1 1

C	-0.20619500	0.32296000	-0.44122300
N	1.09531800	0.88302900	-0.37012800
C	-1.15504400	1.33439400	-0.54757000
C	-0.55859000	-1.02851600	-0.41740200
C	0.99233300	2.35099000	-0.21321100
C	2.23960900	0.21087300	-0.45490000
C	-0.48686000	2.68586300	-0.57608800
C	-2.50799500	1.01332000	-0.63891300
C	-1.90965200	-1.35084100	-0.52086500
C	1.32135100	2.76062800	1.22545700
C	3.51782100	0.76882000	-0.37123800
C	-2.89276100	-0.33150200	-0.63638200
O	-2.36077000	-2.63771300	-0.51393400
O	1.41453500	2.00420900	2.16827700
C	4.69665700	0.03602200	-0.51506500
O	-4.17857300	-0.77656000	-0.73733500
C	4.72602700	-1.44266300	-0.85060700

C	5.94782000	0.69575300	-0.45655800
C	-5.21316900	0.14068400	-1.09732500
C	5.99626600	-2.13287800	-0.32809400
C	7.12877300	-0.01232200	-0.60569100
C	-6.42955500	-0.69860600	-1.52404200
O	-5.50267100	1.07632100	-0.08489100
N	7.17208300	-1.35437800	-0.66782900
C	5.88733700	-2.43290600	1.18309500
C	8.47438200	0.64755700	-0.70725900
C	-7.19415700	-1.28953700	-0.31861900
O	-7.28787800	0.10847500	-2.33044300
C	-6.28556800	0.69681200	1.07351900
O	7.03654500	-2.24079600	1.84239000
O	4.86883000	-2.85142400	1.69488900
O	8.41212100	1.98144300	-0.66732000
O	9.50322900	0.00556500	-0.81728400
C	-7.49900000	-0.17732300	0.71181000
O	-6.53762100	-2.40682500	0.25658800
C	-5.43885400	0.21131300	2.24785200
O	-8.47455100	0.72861800	0.14934700
O	-4.93841200	-1.12004700	2.05868900
H	0.17410000	-1.82181900	-0.31439900
H	1.69070100	2.84687300	-0.88965400
H	2.12300100	-0.85479500	-0.60945900
H	-0.53324800	3.13788000	-1.57183800
H	-0.92448900	3.39750300	0.12795300
H	-3.25231000	1.79707500	-0.69453000
H	3.62426000	1.83380700	-0.19423600
H	-1.61590300	-3.25307100	-0.45077900
H	3.86701200	-1.98331200	-0.45419400
H	4.69823800	-1.54227600	-1.94261300
H	5.99493200	1.77116300	-0.34739300
H	-4.87647600	0.74484000	-1.94523800
H	6.08377700	-3.11287500	-0.80726600
H	-6.07361000	-1.51551700	-2.15620800
H	8.08466000	-1.79466000	-0.70263500
H	-8.15078900	-1.66301800	-0.69985300
H	-7.82222800	0.65901400	-1.72963400
H	-6.69732500	1.65280700	1.41194800
H	6.91106100	-2.49657500	2.77431200
H	9.31505000	2.34129600	-0.73016700
H	-7.89526600	-0.63005900	1.62765000
H	-5.83497400	-2.09130400	0.86384500
H	-4.60249600	0.90755200	2.37767100
H	-6.06965800	0.25298400	3.14349200
H	-9.34028500	0.29460400	0.14536600
H	-4.69733500	-1.49470900	2.91664900

O	1.47061600	4.09144400	1.30158000
H	1.63873600	4.34097500	2.22839100

Bd

Electronic Energy (a.u.) = -1407,430137

1 1

C	2.97938500	0.09993000	-0.22026600
N	1.77504700	-0.65031600	-0.22607400
C	4.05235800	-0.72125600	-0.55821200
C	3.14002800	1.45706900	0.06910100
C	2.09136300	-2.08842500	-0.36888900
C	0.54380900	-0.15390600	-0.14101200
C	3.57561200	-2.12234300	-0.84494400
C	5.33609800	-0.18637700	-0.62030700
C	4.42585700	1.99066600	0.00229000
C	1.91831400	-2.81206200	0.97011400
C	-0.63387200	-0.90477600	-0.12119000
C	5.52780000	1.16917000	-0.34206000
O	4.70299600	3.29935900	0.26969200
O	1.78895600	-2.27365000	2.04860900
C	-1.91339000	-0.34653600	-0.09494600
C	-2.16451100	1.15029000	-0.16339300
C	-3.04939100	-1.18858100	-0.12763200
C	-3.50454300	1.51258700	0.50070900
C	-4.32733500	-0.65056100	-0.17049400
N	-4.56460700	0.65633500	0.00245000
C	-3.91332200	2.96766600	0.31301500
C	-5.55895300	-1.47270300	-0.42597800
O	-2.92168900	3.80324000	0.64306000
O	-5.01570800	3.31990300	-0.05587600
O	-5.29732900	-2.77230000	-0.59245500
O	-6.66997800	-0.97784600	-0.47619300
H	2.31066000	2.09520400	0.35514800
H	1.42618300	-2.54347500	-1.10488500
H	0.49898300	0.92740700	-0.09348500
H	3.61931700	-2.35273400	-1.91398500
H	4.15102400	-2.88850500	-0.32036800
H	6.18701600	-0.80852900	-0.88351500
H	-0.57543500	-1.98781800	-0.13982700
H	3.89018200	3.77817800	0.48711700
H	-1.37859400	1.72433200	0.32587900
H	-2.17594100	1.44544300	-1.22114000
H	-2.93246900	-2.26172900	-0.19811500
H	-3.40282300	1.35648000	1.58782400
H	-5.51780700	1.00336300	-0.03098500

H	-3.23232000	4.72148200	0.54485200
H	-6.13446300	-3.24508100	-0.74819900
O	1.95368500	-4.13967400	0.78253500
H	1.88285900	-4.58651800	1.64555500
O	6.74979800	1.77243700	-0.37523500
H	7.43286200	1.12950300	-0.61503100

IsoBd
Electronic Energy (a.u.) = -1407,429604

1 1

C	-0.20619500	0.32296000	-0.44122300
N	1.09531800	0.88302900	-0.37012800
C	-1.15504400	1.33439400	-0.54757000
C	-0.55859000	-1.02851600	-0.41740200
C	0.99233300	2.35099000	-0.21321100
C	2.23960900	0.21087300	-0.45490000
C	-0.48686000	2.68586300	-0.57608800
C	-2.50799500	1.01332000	-0.63891300
C	-1.90965200	-1.35084100	-0.52086500
C	1.32135100	2.76062800	1.22545700
C	3.51782100	0.76882000	-0.37123800
C	-2.89276100	-0.33150200	-0.63638200
O	-2.36077000	-2.63771300	-0.51393400
O	1.41453500	2.00420900	2.16827700
C	4.69665700	0.03602200	-0.51506500
O	-4.17857300	-0.77656000	-0.73733500
C	4.72602700	-1.44266300	-0.85060700
C	5.94782000	0.69575300	-0.45655800
C	-5.21316900	0.14068400	-1.09732500
C	5.99626600	-2.13287800	-0.32809400
C	7.12877300	-0.01232200	-0.60569100
C	-6.42955500	-0.69860600	-1.52404200
O	-5.50267100	1.07632100	-0.08489100
N	7.17208300	-1.35437800	-0.66782900
C	5.88733700	-2.43290600	1.18309500
C	8.47438200	0.64755700	-0.70725900
C	-7.19415700	-1.28953700	-0.31861900
O	-7.28787800	0.10847500	-2.33044300
C	-6.28556800	0.69681200	1.07351900
O	7.03654500	-2.24079600	1.84239000
O	4.86883000	-2.85142400	1.69488900
O	8.41212100	1.98144300	-0.66732000
O	9.50322900	0.00556500	-0.81728400
C	-7.49900000	-0.17732300	0.71181000
O	-6.53762100	-2.40682500	0.25658800

C	-5.43885400	0.21131300	2.24785200
O	-8.47455100	0.72861800	0.14934700
O	-4.93841200	-1.12004700	2.05868900
H	0.17410000	-1.82181900	-0.31439900
H	1.69070100	2.84687300	-0.88965400
H	2.12300100	-0.85479500	-0.60945900
H	-0.53324800	3.13788000	-1.57183800
H	-0.92448900	3.39750300	0.12795300
H	-3.25231000	1.79707500	-0.69453000
H	3.62426000	1.83380700	-0.19423600
H	-1.61590300	-3.25307100	-0.45077900
H	3.86701200	-1.98331200	-0.45419400
H	4.69823800	-1.54227600	-1.94261300
H	5.99493200	1.77116300	-0.34739300
H	-4.87647600	0.74484000	-1.94523800
H	6.08377700	-3.11287500	-0.80726600
H	-6.07361000	-1.51551700	-2.15620800
H	8.08466000	-1.79466000	-0.70263500
H	-8.15078900	-1.66301800	-0.69985300
H	-7.82222800	0.65901400	-1.72963400
H	-6.69732500	1.65280700	1.41194800
H	6.91106100	-2.49657500	2.77431200
H	9.31505000	2.34129600	-0.73016700
H	-7.89526600	-0.63005900	1.62765000
H	-5.83497400	-2.09130400	0.86384500
H	-4.60249600	0.90755200	2.37767100
H	-6.06965800	0.25298400	3.14349200
H	-9.34028500	0.29460400	0.14536600
H	-4.69733500	-1.49470900	2.91664900
O	1.47061600	4.09144400	1.30158000
H	1.63873600	4.34097500	2.22839100

Geometry of IsoBd and anions

We present the coordinates of the optimized IsoBd geometries and their respective anions. The other coordinates the geometries optimized are not included, but will be kindly provided after mail request to: ilianaramirez@itm.edu.co

Isobd

O	-2.96669200	3.86941300	0.17986100
O	-5.12537200	3.20425400	0.19291000
O	-5.36327600	-2.82084500	-0.12877100
O	-6.62507200	-0.95603500	0.09509100

O	1.94714800	-2.31528600	1.81230100
O	1.65417000	-4.15726300	0.53661900
O	6.76908900	1.78155300	-0.06518200
O	4.63619600	3.24952900	0.58239500
N	-4.54522900	0.64788600	-0.06531300
N	1.80589100	-0.64209600	-0.31507300
C	-3.80777500	3.00799900	0.03315600
C	-3.48524000	1.58688300	-0.41634300
C	-2.13564900	1.10991700	0.13578000
C	-1.88360400	-0.34572800	-0.21074100
C	-3.01848400	-1.18340000	-0.29648000
C	-4.29653000	-0.67035500	-0.14240200
C	-5.46537100	-1.61293400	-0.06059200
C	-0.60394000	-0.89235300	-0.32725100
C	0.57383900	-0.14841500	-0.22408800
C	1.86214700	-2.96607400	0.64524900
C	2.11691600	-2.06986500	-0.56919700
C	3.62766100	-2.08058800	-0.95774300
C	4.09552900	-0.70596600	-0.55427500
C	5.38609500	-0.18241300	-0.52333000
C	5.55979300	1.14801200	-0.14303800
C	4.45015700	1.95323200	0.20457800
C	3.15960800	1.43347300	0.17801400
C	3.01156000	0.09989700	-0.20920700
H	-2.12550200	1.20649800	1.22970600
H	-1.36377200	1.77247200	-0.25306500
H	-3.41706600	1.63418300	-1.51701200
H	-2.91017200	-2.25356800	-0.41824100
H	-0.54485400	-1.96126200	-0.50381700
H	0.52919400	0.92110600	-0.05907200
H	1.49455600	-2.44326400	-1.38286700
H	4.16997700	-2.88416600	-0.45311200
H	3.73128300	-2.24219700	-2.03516600
H	6.24449400	-0.79200200	-0.78964900
H	2.33026100	2.06879600	0.46600200
H	-5.50270200	0.97579100	-0.02949100
H	-7.35627200	-1.59687600	0.14836400
H	-5.28739200	4.13345100	0.43714500
H	5.58555400	3.45237900	0.56466500
H	1.80987900	-2.94999900	2.53905600
H	7.49810600	1.18763400	-0.29507200

IsobdAC2

O	-2.950909	3.867337	0.117107

O	-5.109394	3.207112	0.161025
O	-5.357176	-2.834854	-0.118369
O	-6.616459	-0.967388	0.082638
O	1.696462	-2.385157	1.851087
O	1.910597	-4.206220	0.518720
O	6.778227	1.763701	-0.053604
O	4.648916	3.238850	0.594177
N	-4.533859	0.641295	-0.033507
N	1.812619	-0.651942	-0.303662
C	-3.792778	3.001313	-0.000859
C	-3.474938	1.568431	-0.412722
C	-2.125782	1.103172	0.152643
C	-1.867182	-0.355433	-0.178166
C	-3.009692	-1.197162	-0.255475
C	-4.284089	-0.685801	-0.117107
C	-5.451703	-1.624712	-0.053655
C	-0.595085	-0.899906	-0.293396
C	0.592156	-0.154338	-0.195119
C	1.875081	-2.970152	0.755874
C	2.123789	-2.089922	-0.523204
C	3.620825	-2.093721	-0.936875
C	4.098200	-0.720641	-0.541576
C	5.389421	-0.197363	-0.510012
C	5.566053	1.132965	-0.130351
C	4.458811	1.941054	0.216920
C	3.167909	1.423790	0.190068
C	3.015418	0.089190	-0.196622
H	-2.128077	1.212593	1.245391
H	-1.352492	1.763984	-0.236664
H	-3.401442	1.589894	-1.514421
H	-2.897671	-2.268371	-0.365517
H	-0.528311	-1.970088	-0.455911
H	0.545716	0.915339	-0.029328
H	1.487106	-2.464637	-1.325737
H	4.166451	-2.896595	-0.435352
H	3.727787	-2.251075	-2.016198
H	6.247816	-0.807962	-0.775140
H	2.340164	2.061358	0.478311
H	-5.492903	0.963595	-0.053309
H	-7.345333	-1.611474	0.123410
H	-5.264281	4.144085	0.378404
H	5.599098	3.437128	0.577660
H	1.809879	-2.949999	2.539056
H	7.504460	1.165129	-0.279892

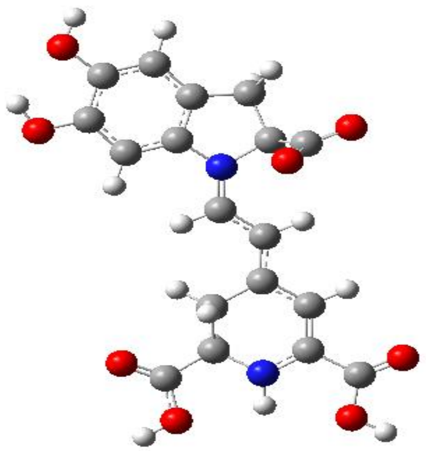

IsobdAC5

O	-2.90066200	3.88196500	-0.17173000
O	-4.96367000	3.28730000	0.52609600
O	-5.33898900	-2.82315000	-0.19469900
O	-6.60285600	-0.96676400	0.06722900
O	1.99217300	-2.34450600	1.79918900
O	1.44054500	-4.09740400	0.48609000
O	6.81479000	1.68754400	-0.05772100
O	4.78952900	3.21511500	0.53522900
N	-4.52369100	0.66019600	0.01548600
N	1.83713800	-0.60705600	-0.31209500
C	-3.87999300	2.99581600	0.05916900
C	-3.48580900	1.59357700	-0.38107800
C	-2.11901700	1.13717700	0.16342200
C	-1.85811900	-0.31625800	-0.19221100
C	-2.99697700	-1.16358900	-0.29018400
C	-4.27472200	-0.67098900	-0.12831100
C	-5.42960900	-1.61195300	-0.09319300
C	-0.58153600	-0.84758700	-0.32162800
C	0.60643600	-0.10575900	-0.20884000
C	1.79093000	-2.94157500	0.61705000
C	2.11603500	-2.04293800	-0.57600100
C	3.63613300	-2.09627800	-0.91179900
C	4.12924900	-0.72577300	-0.52066400
C	5.42851300	-0.24247900	-0.49084500
C	5.66941000	1.11519500	-0.13041500
C	4.50062100	1.93173100	0.19091000
C	3.20951300	1.45305900	0.16489300
C	3.04199200	0.10033100	-0.20001600
H	-2.10534300	1.24064400	1.25675100
H	-1.35290800	1.80153800	-0.23651000
H	-3.41645700	1.62688700	-1.48256800
H	-2.87513200	-2.22949800	-0.43856900
H	-0.51983700	-1.91329900	-0.51937000
H	0.56844400	0.96015500	-0.02363300
H	1.50688900	-2.38505900	-1.41260800
H	4.14370500	-2.90106300	-0.37247800
H	3.77085300	-2.28662400	-1.98125500
H	6.27322200	-0.87803200	-0.74019200
H	2.38134700	2.10228000	0.42780500
H	-5.48045300	0.98937700	0.02208900
H	-7.32203400	-1.62192500	0.09020300
H	-3.20960600	4.76839700	0.08838900
H	5.77242900	3.24663800	0.46838000
H	1.80556900	-2.98346700	2.51081400
H	7.49810600	1.18763400	-0.29507200

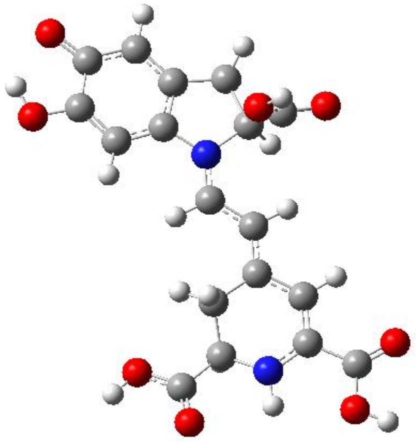

IsobdAC6

O	-2.890422	3.873162	-0.100992
O	-4.970472	3.261491	0.531607
O	-5.348962	-2.818243	-0.205874
O	-6.607555	-0.957658	0.056653
O	1.984001	-2.339443	1.815483
O	1.50729	-4.117846	0.507537
O	6.817411	1.75008	-0.068671
O	4.66713	3.287162	0.536412
N	-4.524238	0.655052	-0.036934
N	1.829733	-0.620465	-0.304937
C	-3.875972	2.984908	0.081600
C	-3.474212	1.593677	-0.388043
C	-2.119335	1.118614	0.167102
C	-1.862588	-0.331121	-0.207714
C	-3.001319	-1.171038	-0.319019
C	-4.278805	-0.669045	-0.158085
C	-5.441896	-1.608940	-0.109223
C	-0.585088	-0.867682	-0.332740
C	0.599477	-0.125915	-0.209679
C	1.819863	-2.950591	0.634481
C	2.131152	-2.049183	-0.560162
C	3.654858	-2.081498	-0.892076
C	4.112895	-0.701366	-0.507026
C	5.402083	-0.160553	-0.471385
C	5.575282	1.175074	-0.123583
C	4.463233	2.063623	0.213780
C	3.169244	1.465089	0.164110
C	3.032425	0.122830	-0.190916
H	-2.117516	1.198480	1.262442
H	-1.344522	1.785814	-0.209277
H	-3.390764	1.651377	-1.487043
H	-2.886111	-2.237116	-0.469284
H	-0.523115	-1.932044	-0.536554
H	0.559623	0.940377	-0.025541
H	1.531475	-2.407789	-1.397339
H	4.169656	-2.876771	-0.343479
H	3.791986	-2.286765	-1.959557
H	6.265854	-0.775924	-0.714919
H	2.320430	2.089004	0.423729
H	-5.475201	0.995709	0.029326
H	-7.332863	-1.605914	0.090971
H	-3.201202	4.753522	0.177392
H	5.585554	3.452379	0.564665
H	1.807550	-2.979940	2.528331
H	7.491102	1.094948	-0.301867

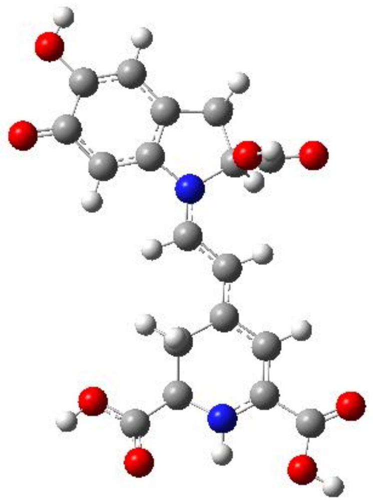

IsobdAC15

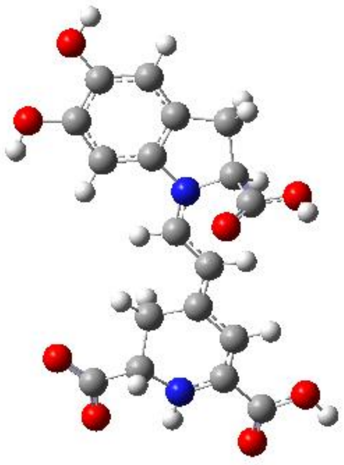

O	-2.983783	3.927542	0.070062
O	-5.12981	3.226354	0.279437
O	-5.409949	-2.748730	-0.056907
O	-6.647008	-0.873879	0.204546
O	1.916076	-2.350130	1.780316
O	1.572998	-4.159428	0.472380
O	6.758948	1.761387	-0.008351
O	4.622718	3.233382	0.629477
N	-4.551393	0.701931	-0.127456
N	1.783203	-0.639521	-0.328305
C	-3.902688	3.072853	0.027582
C	-3.486253	1.646252	-0.444806
C	-2.152825	1.146535	0.114382
C	-1.913654	-0.311693	-0.242955
C	-3.04779	-1.136837	-0.337000
C	-4.328553	-0.607092	-0.158536
C	-5.499772	-1.538525	-0.001240
C	-0.629614	-0.871380	-0.360237
C	0.546415	-0.140518	-0.240461
C	1.811444	-2.975180	0.600119
C	2.087408	-2.062401	-0.596908
C	3.603916	-2.079026	-0.966859
C	4.075225	-0.710075	-0.545330
C	5.367796	-0.192549	-0.496583
C	5.544634	1.133980	-0.103405
C	4.436051	1.939401	0.238914
C	3.142303	1.425437	0.195167
C	2.990231	0.096479	-0.204558
H	-2.143471	1.232350	1.210492
H	-1.369209	1.803080	-0.260642
H	-3.406431	1.691346	-1.544064
H	-2.950493	-2.207909	-0.461231
H	-0.578371	-1.938959	-0.549730
H	0.506649	0.927246	-0.062943
H	1.473331	-2.423357	-1.422860
H	4.135463	-2.890598	-0.463247
H	3.719767	-2.232137	-2.044389
H	6.226107	-0.804081	-0.759454
H	2.313102	2.062877	0.479098
H	-5.465098	1.110642	0.054052
H	-7.373799	-1.513367	0.309012
H	-5.287392	4.133451	0.437145
H	5.572981	3.432024	0.621143
H	1.764659	-2.996771	2.493494
H	7.484972	1.164791	-0.239793

IsobdAC17

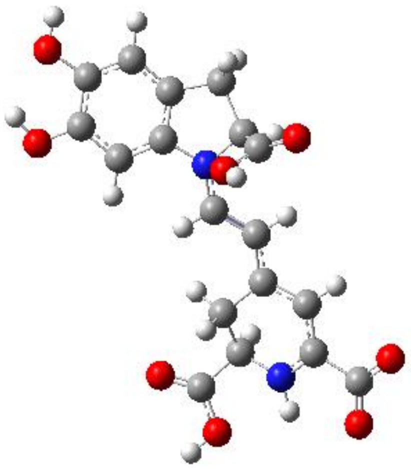

O	-3.030894	3.837521	0.199183
O	-5.184604	3.16079	0.193232
O	-5.39293	-2.840503	-0.070395
O	-6.673767	-0.974906	0.130208
O	1.956426	-2.340856	1.803954
O	1.621522	-4.162278	0.511678
O	6.732181	1.82386	-0.044716
O	4.577136	3.272461	0.590655
N	-4.575979	0.602707	-0.093028
N	1.784872	-0.647652	-0.320223
C	-3.865305	2.971655	0.03437
C	-3.533025	1.560523	-0.436241
C	-2.172983	1.088533	0.09405
C	-1.925565	-0.373637	-0.235999
C	-3.044779	-1.208493	-0.317304
C	-4.339265	-0.703941	-0.146476
C	-5.59181	-1.604124	-0.014765
C	-0.6247	-0.910685	-0.338021
C	0.537561	-0.16392	-0.234576
C	1.848623	-2.974289	0.628025
C	2.105785	-2.06678	-0.577112
C	3.621229	-2.067241	-0.954457
C	4.076695	-0.68802	-0.547315
C	5.362342	-0.152791	-0.509067
C	5.52407	1.17946	-0.128249
C	4.406942	1.971744	0.21184
C	3.11918	1.439954	0.17883
C	2.981695	0.106081	-0.208735
H	-2.137078	1.203012	1.185934
H	-1.407982	1.743380	-0.321316
H	-3.477185	1.624948	-1.536734
H	-2.936889	-2.279154	-0.436318
H	-0.559726	-1.981603	-0.502714
H	0.487261	0.906091	-0.072651
H	1.492696	-2.444573	-1.396617
H	4.165569	-2.867446	-0.446208
H	3.733939	-2.228968	-2.031130
H	6.227400	-0.755343	-0.770903
H	2.283214	2.068972	0.461957
H	-5.553821	0.876508	-0.024723
H	-7.356272	-1.596876	0.148364
H	-5.346870	4.085760	0.452537
H	5.524707	3.483066	0.577112
H	1.817556	-2.985418	2.521493
H	7.464241	1.232490	-0.270137

IbdAN16

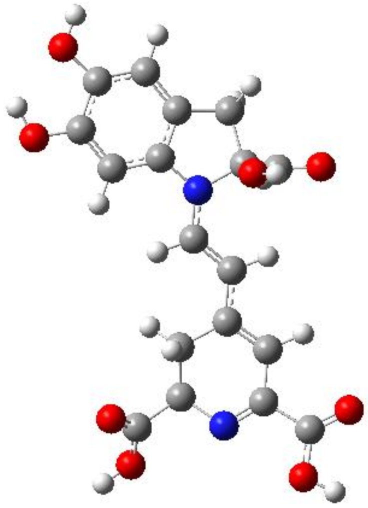

O	-3.47551000	3.46907500	1.08281200
O	-4.79684100	3.56134400	-0.73844500
O	-5.33258200	-2.78772700	-0.05144800
O	-6.65222300	-1.01895800	0.38327600
O	1.98441800	-2.40764600	1.74717900
O	1.52250200	-4.15520100	0.39626700
O	6.76542800	1.82100700	0.01139900
O	4.59867000	3.27118800	0.62393200
N	-4.65601600	0.67106900	-0.12414800
N	1.80980800	-0.63955500	-0.34223300
C	-3.90603000	2.95517900	0.06755800
C	-3.53438200	1.56484300	-0.44254900
C	-2.17444400	1.11084500	0.09573600
C	-1.92851300	-0.34204300	-0.26223100
C	-3.03810800	-1.14415500	-0.37014200
C	-4.35264900	-0.59008200	-0.14666300
C	-5.47593800	-1.58154000	0.06342400
C	-0.60607700	-0.88483800	-0.37460100
C	0.54574000	-0.15238800	-0.25063400
C	1.82239300	-2.98617300	0.54769900
C	2.12136600	-2.04837600	-0.62429000
C	3.64835400	-2.05890700	-0.96167200
C	4.10389100	-0.68312800	-0.53790200
C	5.38940900	-0.15106100	-0.47946100
C	5.55141800	1.17968900	-0.08982800
C	4.43302900	1.96923300	0.23747900
C	3.14214600	1.44045700	0.18487000
C	3.00156300	0.10890700	-0.21004100
H	-2.14113800	1.22390700	1.18787200
H	-1.40429400	1.76992500	-0.31133700
H	-3.48304300	1.62545100	-1.54170600
H	-2.94733700	-2.21123400	-0.53515500
H	-0.54803500	-1.95291300	-0.56440000
H	0.49993000	0.91403500	-0.06440900
H	1.52676200	-2.40555600	-1.46770200
H	4.17458800	-2.86441200	-0.44197800
H	3.78859900	-2.21984600	-2.03540800
H	6.25645900	-0.75469600	-0.73363200
H	2.30477200	2.07238500	0.45754200
H	-5.50270200	0.97579100	-0.02949100
H	-7.30129900	-1.73595000	0.49488700
H	-5.03133400	4.42322000	-0.35003200
H	5.54671500	3.47927300	0.62163200
H	1.81752300	-3.07311600	2.43893600
H	7.49466500	1.22548800	-0.21074400

Algorithms for calculating Riemannian distances are not included, but will be kindly provided after mail justified request to: ilianaramirez@itm.edu.co

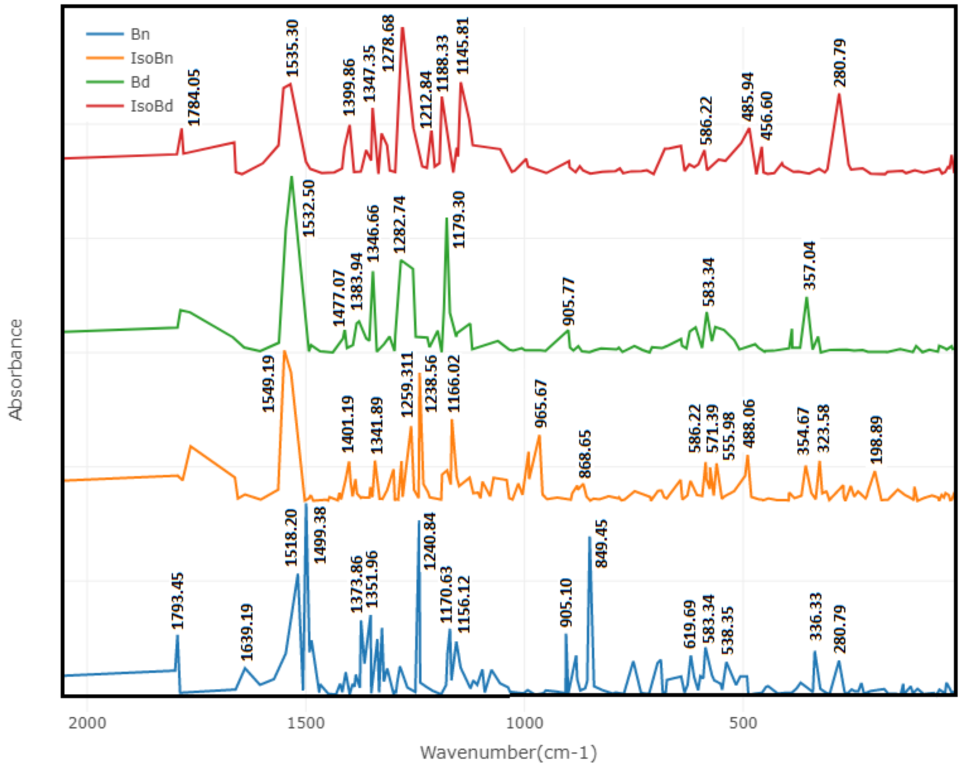

Figure S4. Parent molecules for Betanine (Bn), Isobetanina (IsoBn), Betanidine (Bd), and Isobetanidine (IsoBd).

Table S1. IR data for Bn, IsoBn, Bd, and IsoBd

Assignment	*wavenumber* (cm^{-1})			
	Bn	*IsoBn*	*Bd*	*IsoBd*
C=O antisymmetric stretch; part of double, strong. υ	1793.45			1784.05
C=C stretch, δ	1639.19			
C=C stretch	1518.20	1549.19	1532.50	1535.30
CO COH, δ	1373.86, 1351.96	1401.19, 1341.89	1477.07, 1383.94, 1346.66	1399.86, 1347.35

C-N asymmetric stretching, υ	1240.84	1259.311, 1238.56	1282.74	1278.68, 1212.84
C-C strecting	1170.63, 1156.12	1166.02	1179.30	1188.33, 1145.81
CH2 out-the-plane	905.10,	965.67	905.77	898.21
CH out-of-plane	850			
C=O amidas, out the plane	619.69, 583.34	586.22, 571.39, 555.98	583.34	586.22
	538.35	488.06		456.60
	336.33	323.58, 354.67	357.04	
	280.79	198.89		280.79

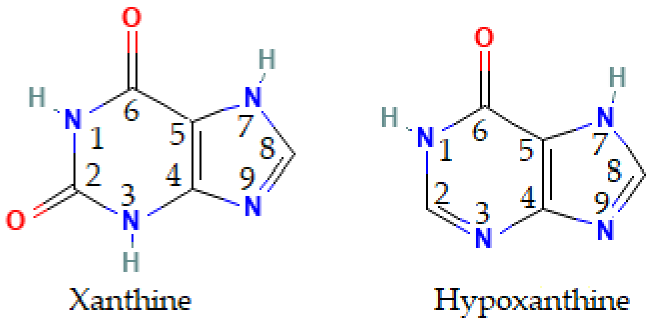

Figure S5. Chemical structures and numbering scheme of xanthine and hypoxanthine

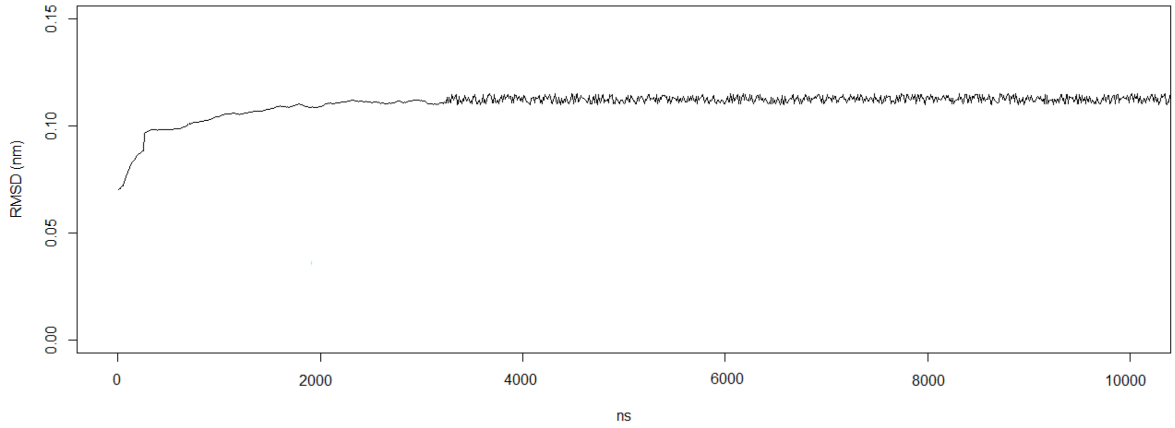

Figure S6. RMSD of the protein backbone during the simulation of DM trajectories
